# Models for Facilitated Transport Membranes: A Review

**DOI:** 10.3390/membranes9020026

**Published:** 2019-02-02

**Authors:** Riccardo Rea, Maria Grazia De Angelis, Marco Giacinti Baschetti

**Affiliations:** Dipartimento di Ingegneria Civile, Chimica, Ambientale e dei Materiali (DICAM), Università di Bologna, Via Terracini 28, 40131 Bologna, Italy; riccardo.rea3@unibo.it (R.R.); grazia.deangelis@unibo.it (M.G.D.A.)

**Keywords:** facilitated transport, permeability models, membrane separation, CO_2_ capture, gas separation, modeling

## Abstract

Facilitated transport membranes are particularly promising in different separations, as they are potentially able to overcome the trade-off behavior usually encountered in solution-diffusion membranes. The reaction activated transport is a process in which several mechanisms take place simultaneously, and requires a rigorous theoretical analysis, which unfortunately is often neglected in current studies more focused on material development. In this work, we selected and reviewed the main mathematical models introduced to describe mobile and fixed facilitated transport systems in steady state conditions, in order to provide the reader with an overview of the existing mathematical tools. An analytical solution to the mass transport problem cannot be achieved, even when considering simple reaction schemes such as that between oxygen (solute) and hemoglobin (carrier) (A+C⇄AC), that was thoroughly studied by the first works dealing with this type of biological facilitated transport. Therefore, modeling studies provided approximate analytical solutions and comparison against experimental observations and exact numerical calculations. The derivation, the main assumptions, and approximations of such modeling approaches is briefly presented to assess their applicability, precision, and flexibility in describing and understanding mobile and fixed site carriers facilitated transport membranes. The goal is to establish which mathematical tools are more suitable to support and guide the development and design of new facilitated transport systems and materials. Among the models presented, in particular, those from Teramoto and from Morales-Cabrera et al. seem the more flexible and general ones for the mobile carrier case, while the formalization made by Noble and coauthors appears the most complete in the case of fixed site carrier membranes.

## 1. Introduction

In membrane separation processes, the term “facilitated transport” (FT) refers to a coupled transport mechanism composed of two main effects: pure physical diffusion and reversible chemical reaction between the target solute (A) and the carrier (C). At the feed side of the membrane, the solute reacts and the reaction product (AC), also referred to as “complex” in the usual terminology of FT, diffuses across the thickness. At the downstream side, the solute is released by the inverse chemical reaction. The unreacted solute, as well as the other dissolved species (that do not interact chemically with the carrier) merely diffuse in the system according to Fick’s law. This carrier-mediated transport leads to a superior performance in terms of solute flux and separation ability of the membranes [1,2].

Based on the mobility of the carrier species, there are two main families of carrier-mediated membranes: the mobile carrier (MC) systems and the fixed site carrier (FSC) ones. In the first case the carrier is free to diffuse across the membrane (supported or immobilized liquid membranes), in the second one the carrier agent is fixed, or bounded, to the polymeric backbone. In this latter case, the complex diffuses following a more complex mechanism than the one in the mobile carrier systems. This point will be further explained in the section referring to transport in FSC membranes.

In 1940, Osterhout et al. [3], firstly investigated systematically the molecular carrier mechanism acting on the transport of potassium and sodium ions in human body, opening a new frontier. After that, in the early 1960s, Scholander [4] and Wittemberg [5] recognized, separately, the facilitation effect that hemoglobin (Hb) and myoglobin (Mg) had on the oxygen transport in blood. They concluded that Hb and Mg react reversibly with the oxygen and diffuse across a liquid film as oxy-Hb or oxy-Mb while, at the opposite side, the oxygen is released and the carrier re-formed.

Starting from those results, many subsequent studies dealt with facilitated transport in membranes. In particular, many works were devoted to the understanding of the principles behind the mechanism, on the key parameters governing it, as well as on the effect of such parameters on the separation performances [6,7,8,9].

The compactness, energy-efficiency, and ease of operation of membrane systems make facilitated transport membranes one of the most competitive and promising technologies in industrial separations [10,11,12]. In this context, great interest is focused on some typical separations of the chemical industry.

Olefin and paraffin separation by facilitated transport membranes (FTM) are reported since the 1980s. In this field, the commonly used carrier is a solution of silver nitrate, usually supported on a polymer matrix, that reacts selectively with the pi-electrons of olefins. In this field of application, Teramoto et al. [13] reported in 1986 results obtained for the ethylene/ethane separation, while Ho and co-worker, in 1994, studied the butene and isobutene separation from butane and propane, respectively [14]. One year before, Funke et al. [15] used hydrated Nafion as polymeric network in enhanced cis and trans 2-butene transport. A review of olefin/paraffin separation using FTM has been published in 2012 by Faiz and Li [16]. More recent works dealt with the use of N-methyl pyrrolidone coupled with AgNO_3_ salt as liquid membranes, supported on tetrafluoroethylene (PTFE), by Azizi et al. [17] to separate propylene from propane in 2015. Supported ionic liquid membranes have been recently used by Zarca and co-workers [18] to enhance the selectivity of propylene/propane. FSC systems based on polymer incorporating silver nanoparticles are under study in olefin separation, to overcome the chemical stability issues of the silver ion [19,20,21,22,23].

Metal complexes have been extensively studied in FSC membranes to facilitate the transport of oxygen. In the 1986–87 two different papers were published, Okahata et al. [24] and Nishide et al. [25], about the facilitation effect in oxygen separation membranes made by cobalt complex, CoPIm, chained to poly-butyl methacrylate polymer. Two years later [26] Ohyanagi and co-workers investigated the change of polymer matrix and metal complexes on the oxygen transport by using FePIm as fixed carrier in poly-methyl methacrylate membrane. Poly-carbonate/COSALEN cobalt complex systems was used in 1996 by Chen and Lai [27], picket-fence Cobalt porphyrin was englobed in different polymer matrix by Shentu and Nischide in 2003 [28]. Preethi et al. [29] complexed the poly vinyl imidazole with cobalt-pthalocyanine in 2006 and studied the effects on the reversible complexation with the oxygen. Three years ago, in 2016, Choi et al. [30] prepared hollow fibers made by poly-ether sulfone and poly-dimethyl siloxane and cobalt-tetraphenylporphyrin (CoPTT) and analyzed the possibility to use these systems to separate oxygen in post-combustion as flue gas pre-treatment to remove CO_2_ before the scrubber unit.

A large number of studies deals with the CO_2_ separation from other gases, such as N_2_ (post-combustion), CH_4_ (biomethane and natural gas upgrading), H_2_ (steam reforming purification) [31,32,33]. The massive anthropogenic emissions of the last decades are changing dramatically the environment in terms of global temperature raise, oceans acidification, as well as Arctic and Antarctic defrost and sea level rise [34]. Facilitated transport membranes could be an interesting, promising, ecofriendly alternative to the common solutions, such as amino based-separations unity. Guha et al. [35] in 1990 used an aqueous solution of diethanol amine (DEA) immobilized in poly-propylene microporous support, Davis and Sandall also used DEA aqueous solution but immobilized in poly-ethylene glycol, in 1993 [36]. Teramoto, in 1996, used poly-vinyl diene difluoride as supporting polymer for DEA and monoethanol amine (MEA) solutions [37]. FSC membranes have been extensively tested in the CO_2_ separation in recent years. Matsuyama et al. used poly-ethylene imine blended with poly-vinyl alcohol [38], and Ho and co-workers studied mobile-fixed hybrid carrier systems made by poly-allyl amine/poly-vinyl alcohol blend with amino acid and potassium hydroxide in 2006 and 2008, [39,40]. Deng et al. [41] presented their results about the facilitated transport of carbon dioxide in poly-vinyl amine and poly-vinyl alcohol blend membranes in 2009. Poly-vinyl amine membranes were also tested in a pilot scale plant in 2013 by the same research group [42] showing stability of performance over four months of test and another pilot scale test was conducted in 2017 and reported here, [43]. Nowadays, concerning FTM for CO_2_ separations, the hybrid systems containing nanofillers [44,45,46] as well as mobile and fixed sites carrier, such as ionic liquids or amino acid salts or polymers containing suitable pendant groups, [47,48,49,50,51,52] are investigated to increase membrane stability and at the same time to obtain high selectivity and permeability.

Despite the high number of data reported in literature, especially in the context of FSC systems, there is a general lack of knowledge of the physics underlying the phenomenon. While a great interest is posed on the experimental measurements, the modeling efforts often reduce to finding a fitting curve that interpolates the experimental data. As shown in the next section, the facilitated transport problem, except in some special cases, usually cannot be solved analytically. For the reaction scheme under study in this review, many authors used numerical techniques to solve the differential mass transfer problem. Kutchai et al. [53] used the quasi linearization method to provide the numerical solution and to demonstrate the effect of the physical and chemical parameters on the transport. Ward [54] gave a quantitative explanation of nitric oxide facilitated transport in ferrous chloride solution using Galerkin’s method [55]. Kemena et al. [7] confirmed the presence of an optimal equilibrium constant value, previously hypothesized by Schultz et al. [1], which allows to obtain the maximum facilitation factor as a function of the dimensionless parameters governing the problem. Kirkoppru-Dindi and Noble [56] extended that approach for the multiple sites carrier case. The methods of orthogonal collocation on finite elements, introduced by Carey and Finlayson [57], was used by Jain and Shultz [58] to predict experimental results coming from real cases such as carbon monoxide/hemoglobin solution, carbon dioxide/bicarbonate solution and nitric oxide/ferrous chloride solution. Barbero and Manzanares [59], by the boundary element method procedure of Ramachandran [60], calculated the results for the facilitation factor over the entire range comprise between the chemical equilibrium regime to the pure physical diffusion transport. Basaran et al. [8] extended the work of Kemena et al. [7] to study how the diffusivity ratio between the carrier and the reaction product affects the facilitation. Ebadi Amooghin et al. [61] modeled the CO_2_ facilitated transport in a poly-vinyl alchol (PVA)-amines membrane by the finite element method. Because of the lack of exact analytical solutions, the reaction-augmented transport problem still represents an interesting topic in mathematical and computational studies in the differential nonlinear systems field [59,62,63,64,65,66,67].

The deep investigation by a phenomenological model of this complex transport mechanism, however, could greatly help in developing new and more performing materials, in recognizing the best condition of use as well as in providing useful information for the overall process optimization. The purpose of this review is therefore to provide an insight on the modeling works that, in the last sixty years, various authors developed to model facilitated transport systems. A series of analytical, approximate models are reviewed in this work, starting from the one presented by Friedlander and Keller in 1965 [68] to the one of Zarca et al. [18] proposed in 2017.

For this purpose, the models described in the present work have been divided in two different sections, Mobile Carrier and Fixed Carrier, each of one organized based on the publish date of the single contribution, from the past to the present.

Before that, the common mathematical background is introduced which allows to describe the mass transfer problem in these kind of systems. In this concern, we chose to focus the review on the most used reaction mechanism in the literature, that is:

A+C⇄AC

### Mathematical Background

The carrier-mediated facilitated transport of a gas in a membrane consists of simple diffusion transport, coupled with one or more reversible chemical reactions which allow to transport the target species also in the form of a complex. Such a process is not easy to describe mathematically, because in most cases the differential transport problem is nonlinear and no exact analytical solution exists.

In order to approach the problem, many authors have developed analytical solutions assuming some simplification or approximation such as the attainment of the local equilibrium condition, equal diffusivities coefficients for carrier and complex, and excess of carrier in respect to the solute. These solutions and models proposed will be further investigated and discussed in the next sections.

Here we present the general formulation of the facilitated transport problem in terms of mathematical differential equations expressing the mass balance for the species involved. The treatment will be based on a single chemical reaction in which three species co-exist: solute, carrier, and their reaction product (also called complex).

In a rectangular geometry, for each species *i* involved, we can write the continuity equation:

(1) $$\frac{DC_{i}}{Dt}=\left(\frac{\partial C_{i}}{\partial t}+v_{x}\frac{\partial C_{i}}{\partial x}+v_{y}\frac{\partial C_{i}}{\partial y}+v_{z}\frac{\partial C_{i}}{\partial z}\right)=-\left(\frac{\partial J_{ix}}{\partial x}+\frac{\partial J_{iy}}{\partial y}+\frac{\partial J_{iz}}{\partial z}\right)-r_{i}+r_{i}^{\prime }$$

where:

*C* = concentration
*J* = flux
*r* = dissipative term
*r*’ = generative term
*v* = velocity
*x*, *y*, *z* = cartesian directions
*t* = time

to be considered with consistent units.

From Equation (1), considering the one dimensional problem (*x* direction) in steady state condition, without convective contribution, we have:

(2) $$-\frac{dJ_{i}}{dx}-r_{i}+r_{i}^{\prime }=0$$

To complete the description of the problem, a reaction scheme that describes the chemical interaction between the carrier and the solute species has to be considered. In the present work, the reaction scheme used originally to depict the oxygen facilitated transport by the hemoglobin or myoglobin has been used. Indeed, the facilitated transport modeling was introduced, in the 1960s, to give a theoretical explanation of oxygen enhanced diffusion in hemoglobin and myoglobin solutions experimentally observed [4,5,69,70,71]. Moreover, even in cases where the modeling approaches were not addressed directly to the oxygen transport, the latter case was used as benchmark to test and compare the obtained solutions.

The reaction scheme mentioned, already shown in the introduction, is depicted in Equation (3):

(3) A+C⇄AC

where *A*, *C* and *AC* indicate the solute, the carrier and the carrier complex species respectively. Following this reaction scheme Equation (2) may be rewritten for each component as follows:

(4) $$-\frac{dJ_{A}}{dx}-k_{f}C_{A}C_{C}+k_{r}C_{AC}=0$$

(5) $$-\frac{dJ_{C}}{dx}-k_{f}C_{A}C_{C}+k_{r}C_{AC}=0$$

(6) $$-\frac{dJ_{AC}}{dx}+k_{f}C_{A}C_{C}-k_{r}C_{AC}=0$$

where the forward reaction rate is assumed to depend on both the carrier and solute concentration, while the backward reaction has a linear dependence on the reaction product concentration, *C_AC_*. Both kinetic constants, $k_{f}$ and $k_{r}$, are considered concentration-independent.

The boundary conditions (BC) needed to close the mathematical problem are obtained by assuming that the only species that can enter and leave freely the membrane is the solute. Carrier and carrier complex are considered to remain confined in the membrane, so that no flux of carrier nor reaction product across the boundaries is allowed. In addition, the solute concentration at the interface is generally considered to be in equilibrium with the gas bulk phase, even if this constraint can be removed, as done by Noble et al. [72] which also considered the existence of an external mass transfer resistance.

Based on what discussed, the following boundary conditions holds:

(7) $$x=0\;C_{A}=C_{A}^{0}\;J_{AC}{}^{0}=J_{C}{}^{0}=0$$

(8) $$x=L\;C_{A}=C_{A}^{L}\;J_{AC}{}^{L}=J_{C}{}^{L}=0$$

where L is the membrane thickness. The previous BCs state that the carrier and the reaction product are considered as nonvolatile species and are confined inside the condensed phase.

Since neither the carrier nor the complex can leave the membrane, one has the following integral constraint:

(9) $$\overset{L}{\underset{0}{\int }}\left(C_{C}+C_{AC}\right)dx=C_{T}L$$

where $C_{T}$ is the initial carrier concentration, before it starts to react with the solute.

Now, if we consider that the diffusion mechanism follows Fick’s law for each component, we have:

(10) $$J_{i}=-D_{i}\frac{dC_{i}}{dx}$$

And Equations (4)–(6) can be written as:

(11) $$D_{A}\frac{d^{2}C_{A}}{dx^{2}}-k_{f}C_{A}C_{C}+k_{r}C_{AC}=0$$

(12) $$D_{C}\frac{d^{2}C_{C}}{dx^{2}}-k_{f}C_{A}C_{C}+k_{r}C_{AC}=0$$

(13) $$D_{AC}\frac{d^{2}C_{AC}}{dx^{2}}+k_{f}C_{A}C_{C}-k_{r}C_{AC}=0$$

And the boundary conditions can now be presented in the form:

(14) $$x=0\;C_{A}=C_{A}^{0}\;\frac{dC_{AC}}{dx}=\frac{dC_{C}}{dx}=0$$

(15) $$x=L\;C_{A}=C_{A}^{L}\;\frac{dC_{AC}}{dx}=\frac{dC_{C}}{dx}=0$$

By the sum of Equations (11) and (13), and integrating twice, it is obtained:

(16) $$D_{A}C_{A}+D_{AC}C_{AC}=C_{1}x+C_{2}$$

where *C*_1_ and *C*_2_ are integration constants, and *C*_1_ in particular represents the total solute flux.

Similarly, adding Equations (12) and (13), we have, for the carrier:

(17) $$D_{C}C_{C}+D_{AC}C_{AC}=C_{3}x+C_{4}$$

where now $C_{3}$ represents the total carrier flux, however by using the boundary Conditions (14) or (15) we can see that such flux is 0 at the boundaries and therefore, being $C_{3}$ a constant, it is zero everywhere.

In the case of mobile carrier, then, by considering the carrier and complex diffusivities equal ($D_{C}=D_{AC}=D$), the Equation (17) can be rewritten as:

(18) $$C_{C}+C_{AC}=C_{5}$$

where $C_{5}$ = $C_{4}/D$.

Moreover, in this case, with the integral Constraint (9) it can be shown that at any point in the membrane:

(19) $$C_{C}+C_{AC}=C_{5}=C_{T}$$

In the case of fixed carrier, on the other hand, the diffusion coefficient $D_{C}$ can be considered as zero while $D_{AC}$ now is an effective diffusion coefficient associated to a sort of jumping mechanism of *A* from one fixed site to the other.

The solute, in the facilitated transport process, is transferred from one boundary to the other by two different mechanisms, the pure diffusion in unreacted state, and the diffusion as a complexed species. Once on the downstream side of the membrane, the reverse reaction takes place and the solute is released. Hence, the total solute flux is expressed as:

(20) $$J_{A}=-D_{A}\frac{dC_{A}}{dx}-D_{AC}\frac{dC_{AC}}{dx}$$

The ratio between the total flux of A inside the membrane, Equation (20), and the flux given by the pure diffusion mechanism leads to the definition of the facilitation factor *F*:

(21) $$F=\frac{-D_{A}\frac{dC_{A}}{dx}-D_{AC}\frac{dC_{AC}}{dx}}{-D_{A}\frac{dC_{A}}{dx}}$$

The facilitation factor is a direct measure of the effect of the reaction on the transport. It easy to see that, if no chemical reaction occurs or the facilitation factor is equal to one, we fall in the pure physical diffusion case.

The set of equations given above, Equations (11)–(13), together with the boundary Conditions (14) and (15), compose a system of nonlinear differential equations that cannot be exactly solved by analytical methods.

In some particular conditions, however, the problem can be easily solved and the analytical solution found. This is the case when two asymptotic conditions are satisfied, that are: (i) chemical equilibrium among the species throughout the membrane thickness (fast chemical reaction) and (ii) pure physical diffusion (negligible effect of chemical reaction). Olander [73] was the first who solved the facilitation by using the chemical equilibrium assumption. In this regime, the overall reaction rate is zero, and the equilibrium constant of the reaction, in terms of concentrations, is given by:

(22) $$K_{eq}=\frac{C_{AC}}{C_{A}C_{C}}$$

In the mobile carrier case, if we consider the case of equal carrier and complex diffusivities, using Equation (22) for the chemical equilibrium, the facilitation factor assumes the form of Equation (23):

(23) $$F=1+\frac{K_{eq}C_{T}}{\left(1+K_{eq}C_{A}^{0}\right)\left(1+K_{eq}C_{A}^{L}\right)}\frac{D_{AC}}{D_{A}}$$

The assumption of equal carrier and complex diffusivity was used in most of the models regarding mobile carrier systems reported in this review. This approximation could be explained by considering that the first models for mobile carrier systems were developed to explain how oxygen is transported in hemoglobin solutions. Hemoglobin (carrier) and oxyhemoglobin (reaction product) are very similar in molar volume so this approximation is reasonable for these biological systems [74]. Moreover, this approximation simplifies the integral constraint given in Equation (9) and leads to the Equation (19) for the carrier conservation.

For the fixed carrier case, in which the carrier has zero mobility, this expression, Equation (23), in general it is no longer true also in the equilibrium assumption, but holds in some special cases, as pointed out in the referring section.

For the mobile carrier system the above equation, or the equilibrium condition, represents a good approximation for some real cases of study, as mentioned by Ward [54], Olander [73], Schultz et al. [1,2], and Fatt and La Force [75]. Nevertheless, the assumption of chemical equilibrium leads to an expression for the complex and carrier concentration that cannot satisfy the boundary conditions. In fact, the reaction product is correlated to the solute concentration by:

(24) $$C_{AC}=\frac{K_{eq}C_{T}C_{A}}{1+K_{eq}C_{A}}$$

Differentiating (24) in the x-direction, we have:

(25) $$\frac{dC_{AC}}{dx}=\frac{K_{eq}C_{T}}{{\left(1+K_{eq}C_{A}\right)}^{2}}\frac{dC_{A}}{dx}$$

that does not vanish at the boundaries, violating the boundary conditions, since the solute flux is not zero there. Despite this inconsistency, the chemical equilibrium approximation represents a useful limiting case for the modeling of facilitated transport mechanism. Indeed such approximated approach, as shown in the next section, will be used, together with the pure diffusion one, as a starting point for several approximate analytical solution procedures.

## 2. Mobile Carrier Systems

Mobile carrier systems are the most used ones, especially in the carbon capture field. The liquid phase containing the carrier agent ensures high diffusivities of the reaction product, showing better flux performance in respect to the fixed carrier [76,77]. Moreover, in hybrid systems, the fixed sites facilitation effect is usually lower than the mobile one, so that the membranes performance, in terms of flux and selectivity, are more affected by the presence of mobile agents [78]. On the other hand, these systems can undergo chemical degradation, evaporation or wash out of carrier solutions, with consequent loss of separation ability [79]. To avoid these issues, the use of less volatile carrier solutions and/or the use of poly-electrolytes as supporting membranes, that increase the interaction between carrier and polymer phase, represent two methods to improve the membranes stability properties [80,81,82].

### 2.1. Models for Mobile Carrier Facilitated Transport Membranes

In this section a review of several analytical approximate solutions to the facilitated transport problem will be illustrated for the mobile carrier case, from the one of Friedlander and Keller (1965), to that of Morales and Cabrera (2002).

The mass balances, the boundary conditions, and the other mathematical considerations which will be considered hereafter are those already introduced in the mathematical background. For the cases in which they are different from the usual form, we will report the appropriate formulations.

#### 2.1.1. Friedlander and Keller, 1965. Mass Transfer in Reacting System Near Equilibrium: Use of the Affinity Function

In 1965 Friedlander and Keller [68] proposed a solution for the facilitated transport of the solute across a liquid film using the affinity function for reacting system near chemical equilibrium. The reaction rate was assumed linear with activity, and the result was employed in the transport problem across a liquid reactive film. It should be noticed that the present model was derived assuming small variations of (low) concentration across the layer and, for this reason, it is not suitable for systems in which the concentration varies significantly across the thickness.

Near the chemical equilibrium condition, it is possible to linearize the reaction term of the mass flux by means of a Taylor series expansion around zero. By truncating the Taylor expansion at the first order term, the authors approximate the solution for the facilitated factor as:

(26) F=11−1mA∑i1miE(η)

In Equation (26), with $m_{i}$ we indicate the mobility parameter of the *i*-th component.

(27) $$\lambda ={\left[B\left(\underset{i}{\sum }\frac{\upsilon_{i}^{2}}{m_{i}}\right)\right]}^{-1/2}$$

(28) $$\eta =\frac{L}{\lambda }$$

(29) $$E\left(\eta \right)=1-\frac{2}{\eta }\left(\frac{cosh\left(\eta \right)-1}{sinh\left(\eta \right)}\right)$$

η indicates a dimensionless length defined as the ratio between the membrane thickness and a characteristic length λ, given in Equation (27), which is a measure of the ratio between the reaction and the pure diffusive rate transport effects. In the definition of λ, $\upsilon_{i}$ are the stoichiometric coefficients, B is the ratio among the forward reaction rate in the equilibrium condition and RT, where R is the universal gas constant and T the temperature. In dilute conditions, the mobility parameter can be easily expressed as:

(30) $$m_{i}=\frac{C_{i}D_{i}}{RT}$$

and the solution for the facilitation factor is calculated in a straightforward manner.

From Equation (26), it is clear that the reaction always enhances the flux, even though the magnitude of the enhancement is function of the E(η) parameter, Equation (29). Two limiting cases were identified: for η > 100, E(η) is close to unity, and the assumption chemical equilibrium throughout the membrane is reasonable. On the other hand, values of η lower than 1 lead to the pure diffusional case in which the effect of the chemical reaction can be neglected. Furthermore, despite the approximation of near equilibrium condition is assumed throughout the system, the departure from chemical equilibrium is greater close to the boundaries, while it is negligible in the core of the membrane. Indeed, by varying the value of L in Equation (28), one can estimate the minimum thickness required to validate the equilibrium approximation in the actual system conditions.

#### 2.1.2. Blumenthal and Katchalsky, 1969. The Effect of Carrier Association–Dissociation Rate on Membrane Permeation

In 1969 Blumenthal and Katchalsky [83] also provided an approximate analytical solution for systems close to chemical equilibrium. In their work they assumed equal diffusion coefficients for the carrier and the reaction product. In this model, however, the local concentration is allowed to slightly deviate from the equilibrium value, by a departure factor that is function of position. With this assumption, the reaction rate can be expressed as:

(31) $$J_{react}=k_{f}(C_{A}{}^{eq}+\delta_{A})(C_{C}{}^{eq}+\delta_{C})-k_{r}(C_{AC}{}^{eq}+\delta_{AC})$$

where $C_{A}{}^{eq},C_{C}{}^{eq},C_{AC}{}^{eq}$ are the concentration of the various species in the middle of the membrane, considered in equilibrium condition, and taken equal to their mean value across the thickness. Neglecting the second order departure term, Equation (31) becomes:

(32) $$J_{react}=k_{f}C_{A}{}^{eq}\delta_{C}+k_{f}C_{AC}{}^{eq}\delta_{A}-k_{r}\delta_{AC}$$

Differentiating twice the Equation (32) and using Equations (11)–(13) to make the proper substitution, it is possible to write down the following equations:

(33) $$\frac{d^{2}J_{react}}{dx^{2}}=\frac{1}{\lambda^{2}}J_{react}$$

(34) $$\lambda ={\left[\frac{k_{f}C_{A}{}^{eq}+k_{r}}{D_{AC}}+\frac{k_{r}C_{C}{}^{eq}}{D_{A}}\right]}^{-1/2}$$

The parameter λ, expressed by Equation (34), is in principle similar to the characteristic length introduced by Friedlander and Keller [68] and discussed above.

Finally, the authors found the following solution for the reaction rate as function of the distance from the upstream boundary:

(35) $$J_{react}=J_{react}^{0}\left[cosh\left(\frac{x}{\lambda }\right)-coth\left(\frac{L}{2\lambda }\right)sinh\left(\frac{x}{\lambda }\right)\right]$$

$J_{react}^{0}$ is the reaction rate evaluated at solute, carrier and complex concentrations at the upstream membrane side.

From Equation (35) it follows that:

$$J_{react}=-J_{react}^{0}\;x=L$$

$$J_{react}=0\;x=L/2$$

**Figure 1:**
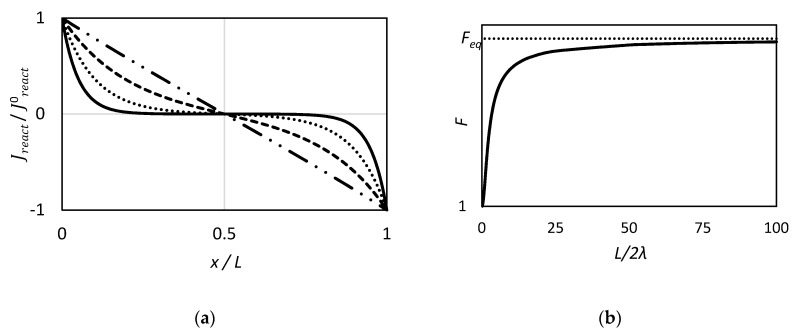
Effect of ratio between membrane thickness (*L*) and characteristic length (*λ*) on the transport in the model of Blumenthal and Katchalsky. (**a**) Size of equilibrium core in the membranes for different *λ* values (Continuous lines *λ* = 0.05, dotted lines *λ* = 0.1, broken lines *λ* = 0.2, broken dotted lines *λ* = 1); (**b**) Facilitation factor as function of *λ*. *F_eq_* represents Equation (40).

In other words Equation (35) states that at the center of the slab the chemical equilibrium region is reached, as the net reaction rate is zero. Moreover, the higher the ratio L2λ, the larger the size of the (near) equilibrium region in the membrane. As one can see in Figure 1a, below, by increasing the value of λ, the equilibrium region becomes smaller, finally being represented by a single point, located in the middle.

The total flux of permeate is expressed as the sum of two contributions, the diffusion in free state and the diffusion in carrier-complex form, according to Equation (20). While usually the concentration of the free species is known at the boundaries, Equations (7) and (8), the reaction product concentration at the boundaries needs to be estimated to calculate the total flux. Blumenthal and Katchalsky derived the analytical solution to calculate the latter, and the solution for the total solute flux becomes:

(36) JA=DAΔCAL+DACKeq−1CT(1+1χ)(Keq−1+CAeq)(Keq−1+CAeq+DACKeq−1CTDA(Keq−1+CAeq)(1+χ))ΔCAL

or, in terms of facilitation factor:

(37) F=1+DACDAKeq−1CT(1+1χ)(Keq−1+CAeq)(Keq−1+CAeq+DACKeq−1CTDA(Keq−1+CAeq)(1+χ))

where

(38) $$\chi =\frac{L}{2\lambda }coth\left(\frac{L}{2\lambda }\right)-1$$

If the membrane thickness is such that L/2λ≫1, Equation (36) can be further simplified. Below we show the flux of solute in these cases for which the local equilibrium condition holds:

(39) $$J_{A}=D_{A}\frac{\Delta C_{A}}{L}+\frac{D_{AC}K_{eq}^{-1}C_{T}}{{\left(K_{eq}^{-1}+C_{A}{}^{eq}\right)}^{2}}\frac{\Delta C_{A}}{L}$$

Thus, the facilitation factor is given by:

(40) $$F=1+\frac{D_{AC}}{D_{A}}\frac{K_{eq}^{-1}C_{T}}{{\left(K_{eq}^{-1}+C_{A}{}^{eq}\right)}^{2}}$$

The differences among Equation (40) and the equilibrium solution reported in Equation (23) come from the approximations used by the authors; they considered a small solute concentration difference between the two boundaries, so that both the upstream and downstream side concentration can be approximated with the mean value in equilibrium condition.

For the opposite case, L/2λ≪1, the parameter χ tends to zero and the latter term in Equation (36) is negligible with respect to the first one, the pure physical diffusion contribution. Obviously in this case the facilitation factor is equal to one, by definition.

In conclusion, the approach of Blumenthal and Katchalsky, although being derived for the near equilibrium conditions, was able to predict the two different limiting case of pure physical diffusion and chemical equilibrium. The ratio between the membrane thickness and the characteristic length, λ, discriminates between the two asymptotic cases.

#### 2.1.3. Goddard et al., 1969. On Membrane Diffusion with Near Chemical Equilibrium Reaction

A different approach was used by Goddard, Shultz and Basset [84] to solve the steady state facilitated transport in liquid membranes. They studied the problem of a fast reaction by using the so called “method of matched asymptotic expansion”, that was already used in many boundary layers problem of transport. By using this method, the spatial domain is divided in three layers: two thin layers close to the boundaries and one thick layer in the middle of the slab. For each layer, a solution is found in terms of power expansion in ϵ terms, which is once again a measure of the importance of reaction terms with respect to the pure diffusion. It is assumed that the middle of the slab is at chemical equilibrium, while in the outer thin layers the reaction and diffusion rate have the same order of magnitude. Since the given solutions are different approximations of the same function, there is a region (overlapping region) in which they are simultaneously valid, and the approximate functions match.

In order to take into account the fast reaction regime, the differential equations, boundary conditions and stoichiometric constraint, Equations (9), (11), (12), (13), (14) and (15) are rewritten by the authors in dimensionless form as follows:

(41) $$\epsilon^{2}\frac{d^{2}\psi_{i}}{dy^{2}}-\mu_{i}\theta =0\;i=A,C,AC$$

(42) $$y=0\;\psi_{A}=\psi_{A}^{0}\;\frac{d\psi_{C}}{dy}=\frac{d\psi_{AC}}{dy}=0$$

(43) $$y=1\;\psi_{A}=\psi_{A}^{L}\;\frac{d\psi_{C}}{dy}=\frac{d\psi_{AC}}{dy}=0$$

(44) $$\overset{1}{\underset{0}{\int }}(\psi_{B}+\psi_{C})dy=1$$

where:

(45) $$\psi_{i}=\frac{C_{i}}{C_{T}}$$

(46) $$\epsilon^{2}=\frac{D_{A}C_{T}}{L^{2}J_{react}^{*}}$$

(47) $$\theta =\frac{J_{react}}{J_{react}^{*}}$$

(48) $$J_{react}^{*}=k_{f}C_{T}^{2}$$

(49) $$\mu_{i}=\frac{D_{A}}{D_{i}}$$

and *y* indicates the dimensionless distance from the upstream boundary, *x/L*. Note that ϵ is qualitatively the ratio between the factor λ, given previously, in Equation (34) and the thickness *L* and that, as said above, it represents a measure of the ratio between the diffusion and the reaction rate.

For the equilibrium core, a power series expansion expresses the concentration profile as function of ϵ:

(50) $$\psi_{i}=\tau_{i}^{\left(0\right)}+\epsilon \tau_{i}^{\left(1\right)}\left(y\right)+\epsilon^{2}\tau_{i}^{\left(2\right)}\left(y\right)+0\left(\epsilon^{3}\right)\;0\left(\epsilon \right)<y<1-0\left(\epsilon \right)$$

In Equation (50), the expansion coefficients $\tau_{i}{}^{j}$ are independent from ϵ. $\tau_{i}^{0}$ reflects the dimensionless equilibrium core concentrations, while the terms multiplied by ϵ represent the departure from the zero reaction rate state, i.e., higher order correction terms of the expansion.

For the reactive zone near the boundaries (0 ≤ *y* < 0(ϵ); 1 − 0(ϵ) < *y* ≤ 1) the following expansions were used:

(51) $$\psi_{i}=b_{i}^{\left(0\right)}\left(\bar{y}\right)+\epsilon b_{i}^{\left(1\right)}\left(\bar{y}\right)+\epsilon^{2}b_{i}^{\left(2\right)}\left(\bar{y}\right)+0\left(\epsilon^{3}\right)\;0\leq y<0\left(\epsilon \right)$$

(52) $$\psi_{i}=c_{i}^{\left(0\right)}\left(\underline{y}\right)+\epsilon c_{i}^{\left(1\right)}\left(\underline{y}\right)+\epsilon^{2}c_{i}^{\left(2\right)}(\underline{y})+0\left(\epsilon^{3}\right)\;1-0\left(\epsilon \right)<y\leq 1$$

where:

$$\bar{y}=\frac{y}{\epsilon }$$

$$\underline{y}=\frac{\left(1-y\right)}{\epsilon }$$

The two “strained” coordinates $\bar{y}$ and $\underline{y}$ are used to define the limits of the boundary regions, starting from the upstream and downstream boundaries to the internal core respectively.

Even in this case, the core equilibrium length is strictly correlated to a parameter that relates the diffusion and reaction contribution to the transport, ϵ. The lower the value of ϵ, the thinner the boundary regions and, thus, the thicker the equilibrium core. According to the asymptotic matching technique [85], the internal core solution (50) has to match, for *y* → 0(ϵ) and *y* → 1 − 0(ϵ), with the solution for the reactive boundaries, Equations (51) and (52), for $\bar{y}$ → ∞ and $\underline{y}$ → ∞ respectively.

The final solution was presented, for the case in which $D_{C}=D_{AC}$, in terms of ratio between actual facilitation factor and facilitation factor in the equilibrium regime, *F*/*F_eq_* truncated to the first term of the expansion:

(53) $$\frac{F}{F_{eq}}=1-\left[G\left(\bar{Z}\right)+G\left(\bar{Z}\right)\right]\sigma^{1/2}\epsilon$$

where *F_eq_* is the one derived in the mathematical background and hereafter again shown:

$$F_{eq}=1+\frac{K_{eq}C_{T}}{\left(1+K_{eq}C_{A}^{0}\right)\left(1+K_{eq}C_{A}^{L}\right)}\frac{D_{AC}}{D_{A}}$$

The functions required to calculate the facilitated factors, G(Z), as function of $\bar{Z}$, $\bar{Z}$ and σ are reported in the Supplementary Materials (SM).

This model showed a good agreement with the numerical calculations of Kutchai et al. [53] regarding the facilitated transport of oxygen in hemoglobin solutions for three different membrane thicknesses, but the model becomes less accurate as thickness decreases. Moreover, in some situations, when the facilitated equilibrium factor is too large, the present method, truncated at first order in ϵ, is unable to give quantitative agreement. In those cases, the authors suggest to use the present solution as a tool to estimate qualitatively the departure from equilibrium of the system of interest.

#### 2.1.4. Kreuzer and Hoofd, 1970. Facilitated Diffusion of Oxygen in the Presence of Hemoglobin

In 1970, Kreuzer and Hoofd [86] derived a set of analytical equations which allow to calculate the total permeate flux and to describe the concentration profiles in facilitated transport systems by considering the same diffusivity for carrier and complex. In this work, the high solute concentration side is located at *x* = *L*.

The solution was still found considering a zone close to the boundaries where there is no chemical equilibrium. However, according to the authors, such region is reasonably small and the carrier concentration therein should not differ much from its boundary values, due to the surface impermeability Conditions (14) and (15). This approximation will be removed in a subsequent work by the same authors in 1972 [87], discussed in the following. In the middle of the slab, on the other hand, the near equilibrium condition was employed to derive the solution.

As mentioned, in the region close to the boundaries it is reasonable to make some assumptions. For *x* close to zero, i.e., in the low solute concentration side, the carrier concentration is approximately constant and very similar to its boundary value. On the opposite side, the same assumption holds. By using these considerations, the system of differential equations governing the problem can be solved for the concentration and, ultimately, for the total flux.

Equations (54) and (55) report the solutions derived for the solute and complex concentrations for *x* → 0.

(54) $$C_{A}=k_{r}\left(\frac{J_{A}x+B}{\alpha^{2}D_{A}D_{AC}}\right)-D_{AC}E_{1}e^{-\alpha x}\;x≅0$$

(55) $$C_{AC}=k_{f}C_{C}^{0}\left(\frac{J_{A}x+B}{\alpha^{2}D_{A}D_{AC}}\right)-D_{A}E_{1}e^{-\alpha x}\;x≅0$$

where α, defined below, is a parameter similar in meaning to $\lambda^{-1}$ in Equation (34).

(56) $$\alpha =\sqrt{\frac{k_{f}C_{C}^{0}}{D_{A}}+\frac{k_{r}}{D_{AC}}}$$

Similarly, for the region close to other boundary, we have:

(57) $$C_{A}=k_{r}\left(\frac{J_{A}x+B}{\beta^{2}D_{A}D}\right)+D_{AC}H_{1}e^{-\beta \left(L-x\right)}\;x≅L$$

(58) $$C_{AC}=k_{f}C_{C}^{0}\left(\frac{J_{A}x+B}{\alpha^{2}D_{A}D_{AC}}\right)-D_{A}H_{1}e^{-\beta \left(L-x\right)}\;x≅L$$

where β is the same as α but calculated at the opposite limiting surface:

(59) $$\beta =\sqrt{\frac{k_{f}C_{C}^{L}}{D_{A}}+\frac{k_{r}}{D_{AC}}}$$

The constants *H*_1_, *E*_1_, and *B* are reported in the supporting materials.

By differentiating twice Equations (54) and (57) it is easy to recognize an exponential decay (growth) of solute concentration with the distance from the boundary located at *x* = *L* (*x* = 0). This suggests the presence of an equilibrium region inside the membrane where the concentration could be considered constant.

With the assumption of local equilibrium in the middle of the slab, the following equations allow to evaluate the boundaries concentration of the reaction product, $C_{AC}^{0}$ and $C_{AC}^{L}$:

(60) $$C_{AC}^{0}=\frac{k_{f}C_{T}\left(C_{A}^{0}+E\right)}{k_{r}+k_{f}\left(C_{A}^{0}+E\right)}+\frac{D_{A}}{D_{AC}}E$$

(61) $$C_{AC}^{L}=\frac{k_{f}C_{T}\left(C_{A}^{L}-H\right)}{k_{r}+k_{f}\left(C_{A}^{L}-H\right)}-\frac{D_{A}}{D_{AC}}H$$

where $E=D_{AC}E_{1}$, $H=D_{AC}H_{1}$.

For the total flux, after some algebra, it is possible to derive the following formulas:

(62) $$J_{A}L=\left(C_{A}^{L}-H-C_{A}^{0}-E\right)\left\{D_{A}+\frac{D_{AC}k_{f}k_{r}C_{T}}{\left(k_{r}+k_{f}C_{A}^{L}-k_{f}H\right)\left(k_{r}+k_{f}C_{A}^{0}+k_{f}E\right)}\right\}$$

(63) $$J_{A}=\alpha D_{A}E\left\{1+\frac{D_{A}{\left(k_{r}+k_{f}C_{A}^{0}+k_{f}E\right)}^{2}}{D_{AC}\left(k_{f}k_{r}C_{T}\right)}\right\}$$

(64) $$J_{A}=\beta D_{A}H\left\{1+\frac{D_{A}{\left(k_{r}+k_{f}C_{A}^{L}-k_{f}H\right)}^{2}}{D_{AC}\left(k_{f}k_{r}C_{T}\right)}\right\}$$

The set of five Equations (62), (63) and (64) plus the two ones for $E_{1}$ and $H_{1}$ (see Supplementary Materials), in five unknowns, $\alpha ,\beta ,H,E,J_{A}$, must be solved via a numerical procedure, in order to calculate the flux for fixed values of the other physical and chemical parameters. By the knowledge of these parameters, it is also possible to calculate the concentration profiles of the species inside the thickness. The equations needed to do that are reported in the original paper, to which we address the reader [86].

If we neglect the departure from the equilibrium condition, Equation (62) collapses on the equilibrium solution for the solute flux, given in Equation (23) in terms of facilitation factor, as one can expect.

The authors tested their calculations against experimental data coming from different authors [5,69,70].

**Figure 2:**
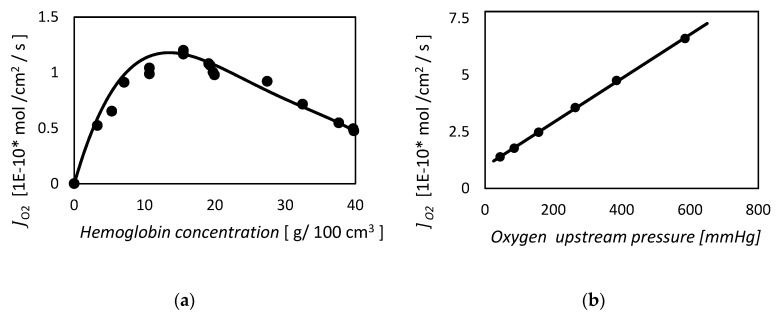
Model of Kreuzer and Hoofd versus experimental data by Wittemberg [5]. (**a**) Oxygen facilitated flux in function of hemoglobin concentration; (**b**) oxygen facilitated flux in function of the oxygen pressure at the feed side. In both figures the points are the experimental data, the line reports the model prediction. The flux reported is the reactive component of the total flux.

In Figure 2 we report the results of the present model when compared with the experimental data of Wittemberg [5] relative to the system of oxygen–hemoglobin. The flux reported in the y-axis is relative to the reactive component of the total solute flux. As we can see, the model agrees very well with the measured data of oxygen in presence of hemoglobin solution impregnated on a Millipore membrane. Moreover, the present approach is able to recognize that the solute concentration gradient is equal for both the downstream and upstream boundary region, suggesting that the pure physical diffusion is a good approximation in this area. However, if tested again the oxygen–myoglobin system, the model is less satisfactory [87].

To improve the prediction performance, after two years, in 1972, Kreuzer and Hoofd published a new analytical approach that was an improvement of the method discussed above [86] by using better approximations in the boundary layers.

#### 2.1.5. Kreuzer and Hoofd, 1972, Factors Influencing Facilitated Diffusion of Oxygen in the Presence of Hemoglobin and Myoglobin

In the work of 1972, the previous description of the membrane region was retained together with the equal carrier and complex diffusivities approximation [87]. However, the assumption of constant carrier concentrations near the boundaries was removed, leading to a better, albeit more complicated, analytical description of the process. As in the previous case, *x* = *L* is the high solute concentration side. Here the boundaries concentrations were evaluated as the difference between the equilibrium contributions and a departure term, function of the distance from the membrane surfaces.

The solute concentration close to boundaries is thus given by:

(65) $$C_{A}=C_{A}^{eq}-\Delta \left(x\right)$$

where the departure function, Δ(x), vanishes far from surfaces.

The carrier and complex concentrations are expressed as follows:

(66) $$C_{C}=C_{C}^{eq}-\frac{D_{A}}{D_{AC}}\Delta \left(x\right)$$

(67) $$C_{AC}=C_{AC}^{eq}+\frac{D_{A}}{D_{AC}}\Delta \left(x\right)$$

The equilibrium concentrations appearing in Equations (66) and (67) are those for carrier, solute and complex evaluated at the two boundaries, between square brackets in Equations (70) and (71), and not the equilibrium ones in the internal core, where the near equilibrium hypothesis is assumed. In other words, the equilibrium concentrations are position-dependent. Inserting Equations (65) to (67) in Equation (1) and using the above mentioned assumptions, the resulting second order differential equation can be solved near the boundaries in term of Δ(x).

The correction term, for *x* → 0 and *x* → *L*, was found to be equal to:

(68) $$\Delta \left(x\right)=\frac{6\alpha_{0}^{2}D_{AC}q_{0}e^{-\alpha_{0}x}}{k_{f}{\left(1+q_{0}e^{-\alpha_{0}x}\right)}^{2}}\;x\to 0$$

(69) $$\Delta \left(x\right)=\frac{-6\alpha_{1}^{2}D_{AC}q_{1}e^{-\alpha_{1}\left(L-x\right)}}{k_{f}{\left(1-q_{1}e^{-\alpha_{1}\left(L-x\right)}\right)}^{2}}\;x\to L$$

where:

(70) $$\alpha_{1}=\sqrt{\frac{k_{f}{\left[C_{C}^{eq}\right]}_{x=L}}{D_{A}}+\frac{k_{r+}k_{f}{\left[C_{A}^{eq}\right]}_{x=L}}{D_{AC}}}$$

(71) $$\alpha_{0}=\sqrt{\frac{k_{f}{\left[C_{C}^{eq}\right]}_{x=0}}{D_{A}}+\frac{k_{r+}k_{f}{\left[C_{A}^{eq}\right]}_{x=0}}{D_{AC}}}$$

The impermeability condition at the boundary for carrier and complex still holds, Equations (14) and (15).

For *x* = 0 and *x* = *L*, considering the previous results, we have the following expressions for the flux:

(72) $$\frac{J_{A}}{D_{A}}{\left[\frac{dC_{AC}^{eq}}{dC_{A}^{eq}}\right]}_{x=0}=\frac{6\alpha_{0}^{3}q_{0}\left(1-q_{0}\right)}{k_{f}{\left(1+q_{0}\right)}^{3}}\left(D_{A}+D_{AC}{\left[\frac{dC_{AC}^{eq}}{dC_{A}^{eq}}\right]}_{x=0}\right)\;x\to 0$$

(73) $$\frac{J_{A}}{D_{A}}{\left[\frac{dC_{AC}^{eq}}{dC_{A}^{eq}}\right]}_{x=L}=\frac{6\alpha_{1}^{3}q_{1}\left(1+q_{1}\right)}{k_{f}{\left(1-q_{1}\right)}^{3}}\left(D_{A}+D_{AC}{\left[\frac{dC_{AC}^{eq}}{dC_{A}^{eq}}\right]}_{x=L}\right)\;x\to L$$

and for the solute concentration at two boundaries:

(74) $$C_{A}^{0}={\left[C_{A}^{eq}\right]}_{x=0}-\frac{6\alpha_{0}^{2}Dq_{0}}{k_{f}{\left(1+q_{0}\right)}^{2}}\;x\to 0$$

(75) $$C_{A}^{L}={\left[C_{A}^{eq}\right]}_{x=L}-\frac{6\alpha_{1}^{2}Dq_{1}}{k_{f}{\left(1-q_{1}\right)}^{2}}\;x\to L$$

The last six Equations (70)–(75) together with the exact solution for the total flux, Equation (76) (integral form of Equation (20)), compose the system of seven equations in the seven unknowns $\alpha_{0}$, $\alpha_{1}$, $q_{0}$, $q_{1}$, ${\left[C_{A}^{eq}\right]}_{x=0}$, ${\left[C_{A}^{eq}\right]}_{x=L}$, $J_{A}$ to be solved, again, with numerical techniques.

(76) $$J_{A}=D_{A}\frac{\left(C_{A}^{L}-C_{A}^{0}\right)}{L}+D_{AC}\frac{\left(C_{AC}^{L}-C_{AC}^{0}\right)}{L}$$

Once the system is solved, the concentration profiles may be determined solving the following equations for $C_{AC}^{eq}$ and $C_{A}^{eq}$:

(77) $$J_{A}x+B=D_{A}C_{A}^{eq}+D_{AC}C_{AC}^{eq}$$

(78) $$B=D_{A}{\left[C_{A}^{eq}\right]}_{x=0}+D_{AC}{\left[C_{AC}^{eq}\right]}_{x=0}$$

Note that in Equations (77) and (78) only equilibrium concentrations appear. As shown in the mathematical background section, they are reciprocally correlated, so that the last two equations can be solved obtaining the species profiles. The model was used by the authors to highlight the influence of characteristic parameters, such as complex diffusivity, backward reaction rate, oxygen back-pressure and actual membrane thickness, on the facilitation factor. However, despite the better agreement reached, in some cases the model predictions remain poor. As pointed out by Jain and Shultz [58], the solutions provided by Kreuzer and Hoofd, even if accurate in describing some systems, becomes progressively less accurate as the Damköhler number drops (as the ‘thin’ film case studied by Smith et al. [88], reported below in this work). In other words, this kind of approach in not suitable for systems in the low facilitation factor regime.

In 1979 [89] the same authors published a revised version of the above approach in which a correction function was used, similarly to Δ in Equations (68) and (69). The correction function was found by solving the problem on the whole volume, instead that on the boundaries only. This fact should improve the quality of the solution. However, the solution procedure has not been extensively explained by the authors, and for such reason it has not been reported in this review.

#### 2.1.6. Yung and Probstein, 1973. Similarity Considerations in Facilitated Transport

A systematic study of the characteristic dimensionless parameters affecting the facilitated transport mechanism was performed by Yung and Probstein in 1973 [90]. For downstream solute concentration equal to zero, they identified three characteristic groups and calculated the numerical solution by varying their values. For some special cases, they also provided analytical solutions which represent the asymptotic behavior of those systems. The approximate solution, hereafter presented, is written in terms of the dimensionless parameters defined below:

(79) $$\kappa =C_{A}^{0}K_{eq}$$

(80) $$\epsilon =\frac{D_{AC}}{k_{r}L^{2}}$$

(81) $$\delta =\frac{D_{A}}{\left(k_{f}C_{T}\right)L^{2}}$$

And the so called ‘disequilibrium parameter’ η is defined as:

(82) $$\eta =\frac{\left(\frac{C_{C}}{C_{C}^{0}}-1\right)}{\kappa }$$

The first parameter presented, κ, in Equation (79), represents the dimensionless equilibrium constant normalized by solute concentration in the upstream side of the membrane. In Equation (80), ϵ is an inverse Damköhler number, i.e., a ratio between the characteristic time of the inverse reaction and the characteristic time of the complex diffusion. δ is an inverse Damköhler number too, but is referred to the diffusion of solute A and to the direct reaction rate, Equation (81).η can be seen as a measure of the departure from the chemical equilibrium condition. Indeed, given the hypothesis of equal diffusivities of carrier and complex and downstream solute concentration equal to zero, it is possible to show that the carrier concentration at the downstream boundary coincides with the total carrier one. Moreover, if the equilibrium condition holds throughout the thickness, the ratio between carrier concentration at the downstream and upstream boundaries is equal to:

$$\frac{C_{C}{}^{L}}{C_{C}^{0}}=1+\kappa$$

(83) $$\frac{J_{A}L}{C_{A}^{0}D_{A}}=1+\Delta \frac{\eta_{L}}{\alpha }=F$$

Hence, the parameter reported in Equation (82), evaluated at *x* = *L* and indicated as $\eta_{L}$, ranging between one, in the equilibrium condition, and zero in the absence of reaction. Equation (83) reports the facilitation factor solution as function of these characteristic parameters, where Δ is the ratio between ϵ and δ, and α is the ratio between the total carrier concentration, $C_{T}$ and the concentration at the upstream boundary, $C_{C}^{0}.$

If no chemical reaction occurs, $\eta_{L}$ is equal to zero and the left side of Equation (83) is equal to 1, corresponding to pure physical diffusion regime.

The mass balances were rewritten using the parameters defined above, and the exact analytical solutions were found for some special cases, i.e., for Δ=0 or κ=0.

The parameter vanishes if the equilibrium reaction constant is << 1 or if the complex diffusivity is negligible with respect to the pure solute one. If the first condition occurs, κ also vanishes. Then, if Δ=0, the solution for η is provided by the following equation:

(84) $$\eta =\eta_{1}\overset{\varepsilon }{\underset{0}{\int }}\frac{1}{\eta_{1}}\left[\overset{\varepsilon^{\prime }}{\underset{0}{\int }}\beta \eta_{1}d\varepsilon^{\prime \prime }\right]d\varepsilon^{\prime }$$

where $\eta_{1}$, the solution to the linear Airy equation, defined in [91].

While, if κ=0, but Δ≠0 (i.e., for low values of $C_{A}^{0}$), the analytical solution is in the form of equation:

(85) $$\eta_{L}=\frac{\left[1-2\sqrt{\tau }tanh\left(\frac{1}{2\sqrt{\tau }}\right)\right]}{\left[1+2\sqrt{\tau }tanh\left(\frac{1}{2\sqrt{\tau }}\right)\right]}$$

where:

$$\tau =\frac{\epsilon }{\left(1+\Delta \right)}$$

**Figure 3:**
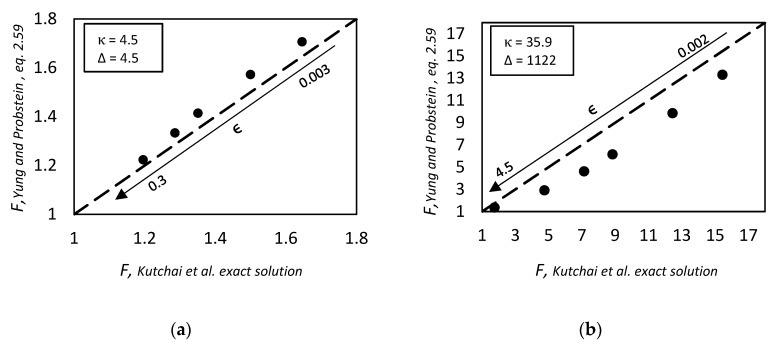
Comparison between facilitation factor F calculated numerically by Kutchai et al. [53] and the value calculated by Equation (86) proposed by Yung and Probstein [90] for different values of ϵ and κ and Δ fixed. (**a**) κ = 4.5, Δ = 4.5; (**b**) κ = 35.9, Δ = 1122. Dashed lines represent the parity condition.

It has been shown that Equation (85) provides excellent agreement with the numerical solution calculated by the same authors, and also with results from Kutchai et al. [53] and Bzdil et al. [92], in a wide range of conditions. At last, also the analytical solution for the facilitation factor, *F*, is presented:

(86) $$F=1+\frac{2\eta_{L}\Delta }{2+\kappa \left(\eta_{L}+1\right)}$$

and tested against the exact values reported by Kutchai et al. [53] for two different sets of κ and Δ by changing ϵ. As we can observe in Figure 3, the approximate solution is in agreement with the exact results. However, for higher values of κ and Δ, as reported in Figure 3b, the discrepancies of Equation (86) become more pronounced, reaching a maximum relative percentage deviation of about 35%, while for low values of these parameters, Figure 3a, the maximum error is 5% in the range investigated.

#### 2.1.7. Smith et al., 1973. An Analysis of Carrier Facilitated Transport

Starting from a magnitude analysis on the governing equations of facilitated transport, in 1973, Smith et al. [88] also provided an approximate analytical solution with the matching of asymptotic expansion technique.

They studied two different cases: the thin film and thick film ones, and found approximate solution for each case. By matching the asymptotic behavior of these, a global solution was found which is able to depict the whole range of thicknesses, approaching the two asymptotes of pure diffusion and chemical equilibrium, respectively. The assumption of equal carrier and complex diffusivities holds in this study.

(1) Thin Films

Based on the governing equations discussed in the mathematical background, one could ensure that the diffusional contribution is predominant over the reactive one in the case of vanishing membrane thickness. However, this condition is also met if the reaction rate is small, compared to the pure diffusion one, so the ‘thin film’ condition is not based on geometric properties only, but, more in general, to the Damköhler number.

For solute and carrier concentrations, and for the total solute flux, the solutions were given as a series expansion, truncated to the second term, containing a dimensionless parameter ϵ, that combines the Damköhler number and equilibrium constant. By using the definition of facilitation factor, the authors derived the following expression:

(87) $$F=\frac{J_{A}L}{D_{A}\Delta C_{A}}=1+\epsilon \gamma_{1}+\epsilon^{2}\gamma_{2}$$

where:

(88) $$\epsilon =\frac{1}{12}\frac{k_{f}C_{T}}{k_{f}\bar{C_{A}}+k_{r}}\left(\frac{k_{r}L^{2}}{D_{A}}\right)$$

(89) $$\Delta C_{A}=C_{A}^{0}-C_{A}^{L}$$

(90) $$\bar{C_{A}}=\frac{C_{A}^{0}+C_{A}^{L}}{2}$$

The terms appearing in the expansions, $\gamma_{1}$, $\gamma_{2}$, are function of the dimensionless length and are reported in the Supplementary Materials.

By knowledge of the physical and chemical parameters, such as the direct and reverse reaction rate, the diffusivities and the total carrier concentration, it is possible to identify the cases in which a first order approximation is accurate for the flux calculation. Indeed, from Equation (87), we see that if $\epsilon \gamma_{1}\gg \epsilon^{2}\gamma_{2}$ the equation reduces to:

(91) $$\frac{J_{A}L}{D_{A}\Delta C_{A}}=F=1+\epsilon \gamma_{1}$$

And it is possible to consider Equation (91), instead of Equation (87), for thicknesses below a threshold value given by:

(92) $$L\ll \sqrt{12\frac{\left(k_{f}\bar{C_{A}}+k_{r}\right)D_{A}}{k_{f}k_{r}C_{T}\gamma_{2}}}$$

(2) Thick Films

When considering thick films, the solution for the concentration profiles is found as a departure from the equilibrium values. For both the carrier and the solute, the equilibrium concentrations were considered not fixed, but rather function of position, indicated here as $\hat{C_{A}}$, $\hat{C_{C}}$. These are to be intended, then, as local equilibrium concentrations. Moreover, even if the local chemical equilibrium could be a reasonable assumption for thick membranes, it does not satisfy all boundary conditions, as shown in the mathematical background section. Therefore, for the departure functions the authors considered that near the surfaces, a boundary layer region is still present where the diffusional effect plays a certain role according to Goddard et al. [84], and Kreuzer and Hoofd [86,87,89].

The concentration profiles are defined as:

(93) $$C_{A}=\hat{C_{A}}+\delta C_{A}\left(x\right)$$

(94) $$C_{C}=\hat{C_{C}}+\delta C_{C}\left(x\right)$$

where the departure functions $\delta C_{A}\left(x\right)$ and $\delta C_{C}\left(x\right)$ are reciprocally correlated by Equation (95):

(95) $$\delta C_{C}\left(x\right)=\frac{D_{A}}{D_{AC}}\delta C_{A}\left(x\right)$$

Near the two boundaries, as already mentioned, the solution is quite different from the one in the core of the system. Here, the perturbation function $\delta C_{A}$ was found to be:

(96) δCA=[CA0−CA^(0)]e−xλ0 xL→0

(97) δCA=[CAL−CA^(L)]e−(L−x)λL xL→1

where λ is a length scale, defined below, and the subscripts indicate the position where the function is evaluated.

(98) $$\lambda ={\left[\frac{k_{f}\hat{C_{A}}\left(x\right)+k_{r}}{D_{AC}}+\frac{k_{f}k_{r}C_{T}}{D_{C}\left(k_{f}\hat{C_{A}}\left(x\right)+k_{r}\right)}\right]}^{-1/2}$$

$\lambda_{0}$ and $\lambda_{L}$ are representative of the thickness of the two existing boundary layers near the membrane surfaces.

The system to be solved via trial and error procedure for the solute equilibrium concentrations at the boundaries, $\hat{C_{A}}\left(0\right)$, $\hat{C_{A}}\left(L\right)$, and for the total flux consists of three equations in three unknowns and is reported hereafter. For the sake of brevity, we address to the original paper for the whole mathematical derivation.

The boundary layers solutions are then used to obtain a solution for the internal zone of the membrane. In fact, the departure function $\delta C_{A}$ above was employed to obtain a perturbation term in the core.

For the internal zone the authors derived a system of equations: hereafter we reported only the one for the flux, dimensionless. The other ones are reported in the Supplementary Materials.

(99) $$J_{A}^{*}=\frac{F}{F^{eq}}=\left\{1-N_{4}G_{0}\left(0\right)-M_{4}G_{L}\left(0\right)\right\}\left\{\frac{1+\frac{F_{eq}-1}{\left[1-N_{3}G_{0}\left(0\right)\right]\left[1+M_{3}G_{L}\left(0\right)\right]}}{F_{eq}}\right\}$$

In the above equation: $\tilde{\lambda_{L}}$ and $\tilde{\lambda_{0}}$ are the parameters defined by Equation (98), calculated for $\hat{C_{A}}\left(L\right)=C_{A}^{L}$ and $\hat{C_{A}}\left(0\right)=C_{A}^{0}$, $F^{eq}$ is the facilitation factor in equilibrium conditions given in Equation (23) and $G_{0},G_{L}$ as well as $N_{3},N_{4},M_{3},M_{4}$, are given in the supplementary section. Also in this case, due to the nonlinearity of the system, a numerical procedure is required to calculate the dimensionless flux.

Finally, for the core, the departure function is found:

(100) $$\delta C_{A}{\left(x\right)}^{core}=2\frac{k_{f}^{2}k_{r}C_{T}J_{A}^{2}}{D_{A}^{3}D_{AC}{}^{2}}$$

The solution method described here provides successful agreement with the numerical calculations of Kutchai et al. [53] in a large variety of cases and for both thin and thick layers.

**Figure 4:**
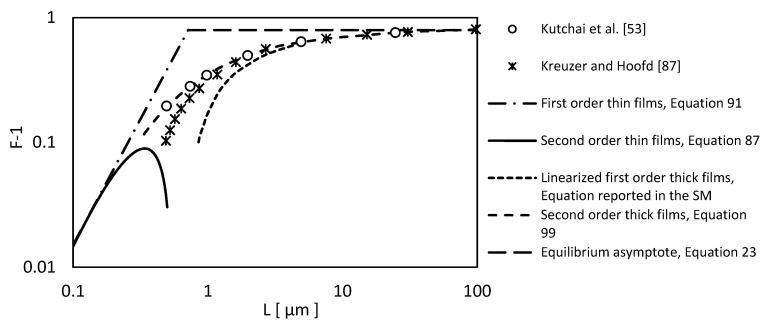
Reactive contribution to facilitation factor as function of membrane thickness.

In Figure 4 we report the model prediction of this approach compared with numerical solution of Kutchai et al. [53] for oxygen transport in hemoglobin solution and, also, with the Kreuzer and Hoofd [87] results. The present method can describe well the solute flux over a wide range of thicknesses, unlike the model Kreuzer and Hoofd [86,87] that does not describe thin films very well.

Despite the higher predictive ability, the method contains many equations and the calculation procedure could be not easy for the thick film case. However, it is worth mentioning that, if the membrane is very thin or very thick, the solution is easily obtained, using Equation (91) or the linearized form of Equation (99) (reported in the Supplementary Materials), respectively.

#### 2.1.8. Smith, Quinn, 1979. The Prediction of Facilitation Factors for Reaction-Augmented Membrane Transport

A straightforward method to calculate the facilitation factor was given in 1979 by Smith and Quinn [93]. Their equation was originally introduced by Donaldson and Quinn [94] as an exact solution to the facilitation transport of CO_2_ in enzymatically bounded polymer in particular conditions. That work has not been discussed in this review, because the reaction scheme used deviates from the one taken as case of study here.

Considering the reaction scheme in Equation (3), the authors, by linearization of the reaction rate, showed that it is possible to obtain an analytical approximate solution that covers the entire regime of kinetic conditions, from fast to slow reaction. For zero downstream solute concentration, with the assumption of equal carrier and complex diffusivities, and considering a constant carrier concentration in the membrane, the reaction rate may be linearized and the solution for the facilitation factor is simply given by:

(101) $$F=\frac{1+X}{1+\frac{X}{\theta }tanh\theta }$$

with:

(102) $$X=\frac{D_{AC}K_{eq}C_{C}}{D_{A}}$$

(103) $$\theta =\frac{1}{2}\sqrt{\frac{k_{f}C_{C}L^{2}}{D_{A}}\left(\frac{1+X}{X}\right)}$$

(104) $$C_{C}=\frac{C_{T}}{1+K_{eq}C_{A}}$$

The Equation (101) can describe quite well the entire range of conditions, from near pure diffusion to near chemical equilibrium condition when an excess of carrier is present. Moreover, for slow chemical reaction rates, the solution may be approximated with the following one:

(105) $$F≅1+\frac{1}{12}\frac{k_{f}C_{C}L^{2}}{D_{A}}$$

Such equation is analogous to the thin film asymptotic solution described in Smith et al. [88]. Indeed, it is easy to recognize from Equations (91) and (105) that, if one assumes the carrier concentration constant and equal to $C_{T}$, and the chemical reaction is very slow, these two equations give exactly the same result.

On the other hand, the opposite case of fast reaction, or chemical equilibrium condition, leads to the following equation for the facilitation factor:

(106) $$F≅1+\frac{K_{eq}C_{C}D_{AC}}{D_{A}}$$

which is the proper equilibrium asymptote for the facilitation factor. By substitution of Equation (104) in (106), for zero downstream side solute concentration, Equation (23) is found.

When we consider the near equilibrium situation, or fast chemical reaction, assuming that the carrier is constant and equal to its equilibrium value, and with the solute concentration set equal to its upstream value, Equation (101) can predict facilitated transport for systems in which the downstream side concentration of solute is negligible. Another case in which the present solution can be used is when a large excess of carrier is employed, as in such case the concentration of carrier is approximatively equal to the total concentration denoted by $C_{T}$. Note that $C_{C}=C_{T}$ requires that $K_{eq}C_{A}^{0}\ll 1$, condition that can be achieved even if the forward chemical reaction is very slow.

**Figure 5:**
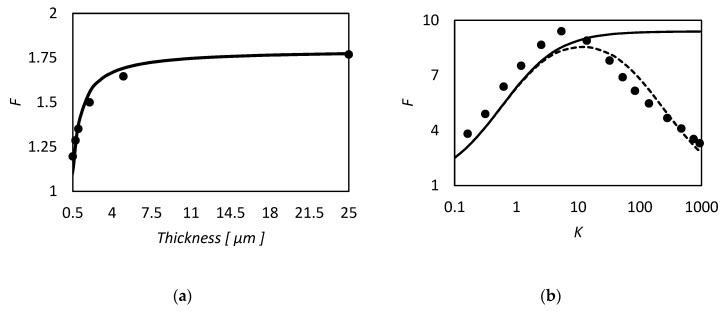
Model prediction of facilitated factor F. (**a**) Numerical results of Kutchai et al. [53] and Equation (101) calculations; (**b**) Asymptotic behavior of Equation (101) as function of dimensionless equilibrium constant (*K* = *K_eq_ C_A_*^0^). Symbols are numerical results from Kemena et al. [7], filled line is Equation (101), dotted line is the improved model of Jeema and Noble, Equation (126).

The discussed solution is mathematically simple, but fails for large constant equilibrium values. Indeed, it is widely recognized that, for high values of *K_eq_* the facilitation effect disappears and *F* tends to unity due to a saturation of the carrier agent (Schultz et al. [1]). This phenomenon implies that there is a value of the equilibrium constant that maximizes the facilitation, as explained in detail by Kemena et al. [7]. On the other hand, in the solution of Smith and Quinn presented here, the facilitation factor reaches a plateau by increasing the equilibrium constant value, as shown in Figure 5.

However in the proper region of validity, the solution of Smith and Quinn represents a powerful tool to provide a prediction of *F*. In Figure 5a we reported as example the prediction of Equation (101) versus the numerical calculation of Kutchai et al. [53] as a function of membrane thickness. The agreement between the two calculations are excellent. On the other hand, Figure 5b shows that the model fails for higher values of equilibrium constant (filled points are the numerical results of Kemena et al. [7]).

#### 2.1.9. Noble et al., 1986. Effect of Mass Transfer Resistance on Facilitated Transport

The external mass transfer resistance was investigated by Noble et al. in 1986 [72] with the aim to avoid the predicted asymptotic behavior of Equation (101). As in the work of Smith and Quinn [93], the case of excess carrier was considered to propose an analytical approximate method.

The solution was presented for the facilitated factor in dimensionless form, using two Sherwood numbers to account for the external resistance to mass transfer. The authors assumed equal diffusion coefficients for carrier and carrier complex and zero downstream solute bulk concentration. It has been found that, for large Sherwood numbers, the results collapse to those proposed by Smith and Quinn [93] and analyzed before.

In the present work, the boundary conditions for the solute are different from those used in previous ones. In particular, to take into account the mass transfer resistance, the boundary conditions are written as:

(107) $$\bar{k_{0}}\left(\frac{C_{A}{}^{0}}{m}-C_{A,0}\right)=-D_{A}\frac{dC_{A}}{dx}\;x=0$$

(108) $$\bar{k_{L}}\left(C_{A,L}-0\right)=-D_{A}\frac{dC_{A}}{dx}\;x=L$$

while for the carrier and the carrier-complex the impermeability surfaces condition still holds.

$\bar{k_{0}},\bar{k_{L}}$ are the mass transfer coefficients in upstream and downstream side respectively, *m* is the partition coefficient (ratio between external phase concentration and membrane phase concentration), $C_{A}{}^{0}$ is the external phase solute concentration in the upstream side, and $C_{A,0}$ and $C_{A,L}$ are the membrane phase concentrations at the upstream and downstream sides respectively.

The facilitation factor is then expressed as:

(109) $$F=\frac{\left(1+\frac{\alpha K}{1+K}\right)\left(1+\frac{1}{Sh_{0}}+\frac{1}{Sh_{L}}\right)}{1+\frac{\alpha K}{1+K}\frac{tanh\left(\lambda \right)}{\lambda }+\left(1+\frac{\alpha K}{1+K}\right)\left(\frac{1}{Sh_{0}}+\frac{1}{Sh_{L}}\right)}$$

where the dimensionless parameters, defined below, according to Kemena et al. [7] and Folkner and Noble [95] represent: a mobility ratio between complex and solute (α), the dimensionless equilibrium constant (K), a parameter that contain the previous three (λ), the Sherwood numbers for the upstream and downstream side ($Sh_{0}$ and $Sh_{L})$.

(110) $$\alpha =\frac{D_{AC}C_{T}m}{D_{A}C_{A}{}^{0}}$$

(111) $$K=K_{eq}\frac{C_{A}{}^{0}}{m}$$

(112) $$\epsilon =\frac{D_{AC}}{k_{r}L^{2}}$$

(113) $$\lambda =\frac{1}{2}\sqrt{\left(\frac{1+\left(\alpha +1\right)K}{\epsilon \left(1+K\right)}\right)}$$

(114) $$Sh=\frac{\bar{k}L}{D_{A}}$$

It is easy to see that, considering the carrier concentration expressed by Equation (115), the solution given here is the same proposed by Smith and Quinn [93], Equation (101), if the Sherwood numbers tends to infinity, and hereafter reported in Equation (116) by using the parameters defined above in Equations (110)–(113).

(115) $$C_{C}=\frac{C_{T}}{1+K_{eq}\frac{C_{A}{}^{0}}{m}}$$

(116) $$F=\frac{\left(1+\frac{\alpha K}{1+K}\right)}{1+\frac{\alpha K}{1+K}\frac{tanh\left(\lambda \right)}{\lambda }}$$

It is equally easy to observe that the lower the Sherwood numbers, the lower the facilitation effect. The main drawback of the external mass transfer resistance are a lower solute concentration in the membrane phase, in the upstream side, and, at the opposite boundary, a higher level of solute. These two effects lead to a reduced driving force for the transport. The present approach was compared with the numerical results calculated by Kemena et al. [7] and the agreement between the approximate solution and numerical calculation was quite good in the range investigated.

#### 2.1.10. Basaran et al., 1989. Facilitated Transport with Unequal Carrier and Complex Diffusivities

A systematic investigation of the effect of diffusivity difference between carrier and complex on the facilitation factor was performed by Basaran et al. in 1989 [8]. Two different asymptotic solutions were found for the two limiting cases of large and small Damköhler number. Those were compared with numerical calculations made by the same authors using the Galerkin finite elements method. It was found that, in general, the ratio between the carrier-complex and the pure carrier diffusivity enhances the facilitated transport effect. They extended the work of Kemena et al. [7] by finding the optimal equilibrium values that maximizes the facilitation also for the case of $D_{C}/D_{AC}\neq 1$.

(1) Small Damköhler Number

For small Damköhler number, the solution for the concentration profiles and facilitation factor was found following the regular perturbation analysis, similarly to what done by Smith et al. [88].

(117) $$P=\frac{k_{r}L^{2}}{D_{A}}$$

The concentration profiles for the solute and the carrier, Equations (118) and (119), are expressed as power expansions in the Damköhler number, defined in Equation (117) and here indicated as *P*.

(118) $$\bar{C_{A}}=\overset{\infty }{\underset{n=0}{\sum }}P^{n}c_{A}^{\left(n\right)}$$

(119) $$\bar{C_{C}}=\overset{\infty }{\underset{n=0}{\sum }}P^{n}c_{C}^{\left(n\right)}$$

$\bar{C_{A}}$ and $\bar{C_{C}}$ are dimensionless concentration, referred to the upstream side, and $c_{A}^{\left(n\right)}$ and $c_{C}^{\left(n\right)}$ are the expansion coefficients. For the calculation of the facilitation factor, the expansion was truncated at the first order for $\bar{C_{C}}$ while, for $\bar{C_{A}}$ the last term considered was the second order.

With this approach, the resulting facilitation factor was presented as:

(120) $$F=1+\frac{1}{12}PKc_{C}^{\left(0\right)}-\frac{1}{720}{\left(PKc_{C}^{\left(0\right)}\right)}^{2}\left\{1+6\frac{ST}{PKc_{C}^{\left(0\right)}T}\left[R+K\left(1+\frac{\bar{C_{A}^{L}}}{2}\right)\right]-\frac{1}{12}\frac{SK}{PT}{\left(\bar{C_{A}^{L}}-1\right)}^{2}\right\}$$

where the zero-th order expansion coefficients appearing, $c_{C}^{\left(0\right)}$, is reported in the equation below:

(121) $$c_{C}^{\left(0\right)}=\frac{T}{1+K\frac{\left(1+\bar{C_{A}^{L}}\right)}{2}}$$

K is the dimensionless equilibrium constant, $K=K_{eq}C_{A}^{0}$, and T=CTCA0.

The other parameters appearing in Equation (120), R and S, are directly correlated to the ratio between the diffusivities of the species by the following relationships:

(122) $$R=\frac{D_{C}}{D_{AC}}$$

(123) $$S=P\frac{D_{A}}{D_{AC}}$$

From Equation (120), one can see that, keeping all the other dimensionless groups fixed, the facilitation factor decreases with increasing R.

(2) Large Damköhler Number

For the large Damköhler number case, the authors found the solution for the flux via a singular perturbation analysis.

The effect of the diffusivities ratio was investigated by linearization of the facilitation factor around *R* = 1, truncated at first order, for the case of the reaction rate is higher than the diffusion one:

(124) $$F=1+{ℑ}_{1}\left(1-\left(\sigma \frac{\eta_{0}\eta_{1}\omega +\rho \sigma \delta }{\eta_{0}\eta_{1}+\sigma \delta }+\frac{\gamma_{0}}{\eta_{0}}+\frac{\gamma_{1}}{\eta_{1}}-2\right)\left(\frac{1-R}{R}\right)\right)$$

In the equation above ${ℑ}_{1},$ reported below in Equation (125), is the facilitation factor in the case of *R* = 1. All the other functions in Equation (124) are reported in the Supplementary Materials.

(125) $${ℑ}_{1}=\frac{\sigma \delta }{\gamma_{0}\gamma_{1}}\left[-\frac{1}{2p_{0}}\left(p_{1}+{\left(p_{1}^{2}-4p_{0}p_{2}\right)}^{1/2}\right)\right]$$

It was shown that:

$$\sigma \frac{\eta_{0}\eta_{1}\omega +\rho \sigma \delta }{\eta_{0}\eta_{1}+\sigma \delta }+\frac{\gamma_{0}}{\eta_{0}}+\frac{\gamma_{1}}{\eta_{1}}-2\leq 0$$

Thus, also in this case, the same dependence of the facilitated factor on the ratio *R* is shown, as for the case of low Damköhler number.

Comparison with numerical calculations done by the authors, shows a qualitatively good agreement between the asymptotic expansion and the numerical solution in both the Damköhler’s number. However, for the near diffusion limit, the approximated solution cannot describe quantitatively the results.

**Figure 6:**
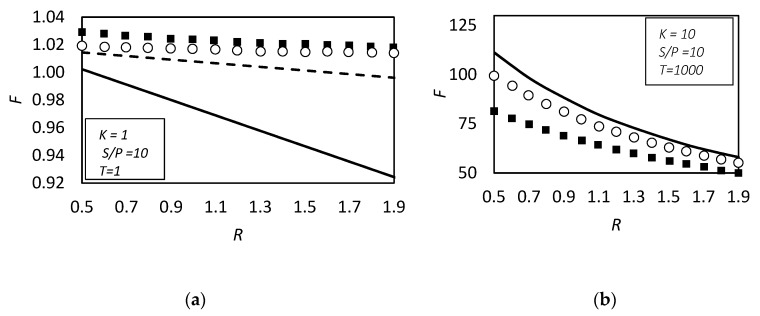
Diffusivity ratio influence on facilitation. (**a**) Small Damköhler number. Black squares and white circles are numerical results for *P* = 1 and 0.5 respectively, full and broken lines are Equation (120) for *P* = 1 and 0.5 respectively; (**b**) Large Damköhler number. Black squares and white circles are numerical results for *P* = 1000 and 10,000 respectively. Full line is Equation (124). In both the case small but nonzero downstream concentration has been considered ($\bar{C_{A}^{L}}$ = 0.01).

Figure 6 shows the two opposite limits. Near the chemical equilibrium condition, Figure 6b, the approximated solution is able to predict the diffusivity ratio effect on the facilitation qualitatively and, moreover, by increasing the Damköhler’s numbers *P* from 1000 to 10,000, the agreement becomes quantitative too. On the contrary, Figure 6a, near the pure diffusion condition the approximated method, even if qualitatively right, can give facilitation factor lower than one, that is impossible by definition (Equation (21)). However, from these results one can conclude that if the system is near the pure physical diffusion, the ratio between carrier and complex diffusivity does not play a significant role in the facilitation.

The dependence of the optimum dimensionless equilibrium constant as function of the Damköhler number was also investigated. As a confirmation of the results already explained, it was shown that for small Damköhler number, the optimum *K* is not affected by the *R* ratio while a pronounced effect appears when the system approaches the fast reaction regime.

#### 2.1.11. Jemaa and Noble, 1992. Improved Analytical Prediction of Facilitation Factors in Facilitated Transport

As already mentioned, the approximate model provided by Smith and Quinn [93] can describe quite accurately the facilitation effect, provided that the dimensionless equilibrium constant is not too high but it is not able to describe the gaussian shape of the facilitation factor as function of the equilibrium constant (Figure 5b). Indeed, for value of *K* higher than the optimal one, the facilitation factor predicted by Smith and Quinn approaches asymptotically a constant value.

To overcome this limitation, Jemaa and Noble [96] proposed an improvement of the approximate solution to take into account a small, but non zero, downstream solute concentration. However, that concentration does not correspond to the actual value, but is rather an adjustable parameter that can be varied to match the analytical approximation to the numerical solution, provided by Kemena et al. [7]. In a graphical way, they reported the adjustable concentration parameter that minimizes the error between the analytical and numerical solution for different operational conditions. A set of optimal dimensionless concentration parameters were calculated for different operative conditions.

The approximate facilitation factor, in the assumption of excess carrier and chemical equilibrium throughout the thickness, and equal diffusivity of carrier and carrier-complex, as in their previous study [72] and in agreement with Smith and Quinn [93], for this case, is:

(126) $$F=\frac{1+\frac{\alpha K}{\left(1+K\right)\left(1+\theta K\right)}}{1+\frac{\alpha K}{\left(1+K\right)\left(1+\theta K\right)}\frac{tanh\left(\lambda \right)}{\lambda }}$$

where the nonzero downstream dimensionless concentration is represented by θ and defined as:

$$\theta =\frac{C_{A}^{L}}{C_{A}^{0}}$$

λ, the composed parameter already defined in Equation (113), is now expressed as:

(127) $$\lambda =\frac{1}{2}\sqrt{\left(\frac{1+\left(\alpha +1\right)K}{\epsilon \left(1+K\right)\left(1+\theta K\right)}\right)}$$

while the other dimensionless parameters α,K and ϵ are still given by the Equations (110), (111) and (112).

Note that for θ equal to zero, Equation (126) is the same as the one reported by Smith and Quinn [93], Equation (116). In Figure 5b above we reported a comparison of the present approach with the exact results of Kemena et al. [7] and with the model results of Smith and Quinn [93] for the parameter set α = 50, ϵ = 0.1 (to use these, refer to Equation (116) instead of (101)). As shown, the present approach has a better predictive ability than the one of Smith and Quinn. Moreover, for non zero actual downstream concentration, Equation (129) could provide a good estimation for the facilitation factor, without any adjustment on the numerical results.

This method could be seen as a simple way to adjust the analytical solution without mathematical complications and provides a good prediction, even if the use of a fitting parameter makes it not fully predictive.

#### 2.1.12. Teramoto, 1994. Approximate Solution of Facilitation Factor in Facilitated Transport

Teramoto in 1994 [74], proposed an analytical approach to describe the facilitated transport for the entire range of Damköhler number, without assuming equal diffusivities or zero solute concentration at downstream side. Starting from the two membrane surfaces, two different facilitation factors were written, the first one from the upstream boundary and the second one from the downstream side. The final solution was found via a trial and error procedure that converges when the two factors match.

For the first case, upstream boundary, it is assumed that the influx could be adequately represented by considering the carrier concentration constant and equal to its value at boundary, an approach very similar to that already used by of Kreuzer and Hoofd [86,87] and discussed above in this work. In analogy, for the second case, the outflux could be express considering the carrier concentration constant and equal to the downstream side value.

For the solute influx, the following boundary conditions were used:

(128) $$C_{A}=C_{A}^{0}\;C_{AC}=C_{AC}^{0}\;\frac{dC_{AC}}{dx}=0\;x=0$$

(129) $$C_{A}=C_{A}^{L}\;\;\;x=L$$

and for the outflux:

(130) $$C_{A}=C_{A}^{0}\;\;\;x=0$$

(131) $$C_{A}=C_{A}^{L}\;C_{AC}=C_{AC}^{L}\;\frac{dC_{AC}}{dx}=0\;x=L$$

The mathematical treatment of Teramoto lead to the violation of some boundary conditions, as it can be seen by comparing Equations (128) to (131) to the ones reported in the mathematical background Equations (7) and (8). According to van Krevelen and Hoftijzer [97], since the solute influx could be more influenced from the carrier concentration at feed side rather than at the downstream, these violations should not dramatically influence the solution for the solute flux through the upstream surface. The same considerations hold for the downstream side.

The set of parameters defined to solve the facilitation problem is:

(132) $$\bar{C_{A}}=\frac{C_{A}}{C_{A}^{0}}$$

(133) $$\bar{C_{AC}}=\frac{C_{AC}}{C_{T}}$$

(134) $$\bar{C_{C}}=\frac{C_{C}}{C_{T}}$$

(135) $$q=\frac{D_{AC}C_{T}}{D_{A}C_{A}^{0}}$$

(136) $$r=\frac{D_{AC}}{Dc}$$

(137) $$\delta =L\sqrt{\left(\frac{k_{f}C_{T}}{D_{A}}\right)}$$

(138) $$\gamma_{0}=\delta \sqrt{\bar{C_{C}^{0}}+\frac{1}{rqK}}$$

and K is the dimensionless equilibrium constant, $K=K_{eq}C_{A}^{0}$.

From the upstream point of view, the facilitation factor was found to be equal to:

(139) $$F_{0}=\gamma_{0}\frac{\left[1+\frac{1}{rqK\bar{C_{C}^{0}}}\right]\left(1-\bar{C_{A}^{L}}\right)+\left(cosh\left(\gamma_{0}\right)-1\right)\left[1-\frac{\bar{C_{AC}^{0}}}{\bar{KC_{C}^{0}}}\right]}{sinh\left(\gamma_{0}\right)+\frac{\gamma_{0}}{rqK\bar{C_{C}^{0}}}}$$

In a similar way, for the opposite boundary, *x* = *L* the facilitation factor is:

(140) $$F_{L}=\gamma_{L}\frac{\left[1+\frac{1}{rqK\bar{C_{C}^{L}}}\right]\left(1-\bar{C_{A}^{L}}\right)+\left(cosh\left(\gamma_{L}\right)-1\right)\left[\frac{\bar{C_{AC}^{L}}}{\bar{KC_{C}^{L}}}-\bar{C_{A}^{L}}\right]}{sinh\left(\gamma_{L}\right)+\frac{\gamma_{0}}{rqK\bar{C_{C}^{L}}}}$$

where:

(141) $$\gamma_{L}=\delta \sqrt{\bar{C_{C}^{L}}+\frac{1}{rqK}}$$

In steady state condition, Equation (139) must be equal to Equation (140). A set of Equations (142), (143), and (144), can be used to express the relationship between the carrier and complex concentrations at the boundaries:

(142) CCL¯=CC0¯+(F−1+CAL¯)q

(143) CACL¯=CAC0¯−(F−1+CAL¯)rq

(144) $$\frac{\left(\bar{C_{AC}^{L}}+\bar{C_{AC}^{0}}\right)+\left(\bar{C_{C}^{L}}+\bar{C_{C}^{0}}\right)}{2}=1$$

Those equations were found by considering sigmoidal and reverse sigmoidal shape of complex and carrier concentrations respectively. The system can be solved to obtain the five unknowns $\bar{C_{C}^{L}},\;\bar{C_{AC}^{L}},\bar{C_{C}^{0}},\bar{C_{AC}^{0}}$ and F via numerical procedure for a given set of values for q,r,K,δ, and $\bar{C_{A}^{L}}$.

Note that the sigmoidal approximation used to express the carrier continuity, as an alternative to the integral constraint given by Equation (9), leads to an algebraic equation, that is easier to use in order to solve the system.

The facilitated factor calculated with the present approximate method was found in excellent agreement, over a wide range of cases studied, with the numerical calculations made by Basaran et al. [8], Kemena et al. [7], and Jain and Schultz [58].

**Figure 7:**
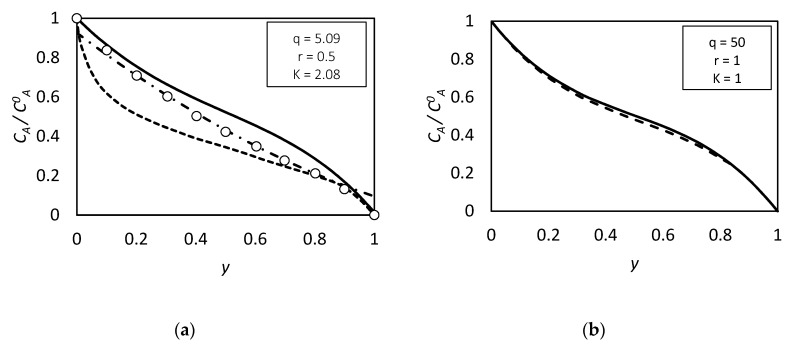
Solute concentration profiles. (**a**) δ = 22.5, circles are numerical solution from Jain and Shultz [58], solid line is the upstream solution, broken line is the downstream solution, dash dotted line is the equilibrium core solution; (**b**) δ = 5, solid line is the upstream solution, broken line is the downstream solution. In both cases the downstream solute concentration is zero.

However, discrepancies arise between the calculated solute concentration profiles (not reported in this work) when δ≫1. In this case, the solutions obtained starting from the different boundaries give different results, as shown in Figure 7a. To improve the concentration profiles accuracy in this case, an equilibrium core in the middle part of the membrane was introduced, and the following equations were given for the middle part of the slab:

(145) $$\left(1-\bar{C_{A}}\right)+q\left(\bar{C_{C}}-\bar{C_{C}^{0}}\right)=Fy$$

(146) $$\left(1-\bar{C_{A}}\right)+rq\left(\bar{C_{AC}}-\bar{C_{AC}^{0}}\right)=Fy$$

Together with the equilibrium condition, and using this approximated method to evaluate $\bar{C_{C}^{L}},\bar{C_{AC}^{L}},\bar{C_{C}^{0}},\bar{C_{AC}^{0}}$ and F Equations (145) and (146) can be used to calculate the concentration profiles. With the last improvement, it is possible describe the solute concentration profile over the entire thickness. On the other hand, if δ is not too large, the concentration profile is well depicted without the equilibrium core correction, Figure 7b.

#### 2.1.13. Morales-Cabrera et al., 2002. Approximate Method for the Solution of Facilitated Transport Problems in Liquid Membranes

An improvement of the method developed by Teramoto [74] was given by Morales-Cabrera et al. in 2002 [98]. Based on the same boundaries approach, the solution, in terms of concentration profiles and facilitated flux, was proposed analyzing the solute flux only at the two boundaries which, at steady state, must obviously be the same. The present method is based on the linearization of the reaction term, using a Taylor expansion truncated at the first order, evaluated at the boundary surfaces.

With respect to the similar approach of Teramoto [74], the approximate solution of Morales-Cabrera et al. consists of a higher number of nonlinear equations, but does not involve simplifications on the carrier continuity equation, Equation (9).

Unlike the other treatments presented, here the *x* = 0 is set in the middle plane of the thickness and the system is split in two subregions. The first region goes from the upstream boundary, *x* = −*L*/2, to the middle plane, *x* = 0, and the second one from the middle plane, *x* = 0, to the downstream boundary surface, *x* = +*L*/2.

The following equations are derived for the dimensionless concentration profiles of the solute in the left region $\bar{C_{A,L}}$, and right region $\bar{C_{A,R}}$:

(147) $$\bar{C_{A,L}}=A_{L}sinh\left(\varphi_{L}y\right)+B_{L}cosh\left(\varphi_{L}y\right)-\frac{\alpha_{L}}{\varphi_{L}{}^{2}}y-\frac{\beta_{L}}{\varphi_{L}{}^{2}}\;-1\leq y\leq 0$$

(148) $$\bar{C_{A,R}}=A_{R}sinh\left(\varphi_{R}y\right)+B_{R}cosh\left(\varphi_{R}y\right)-\frac{\alpha_{R}}{\varphi_{R}{}^{2}}y-\frac{\beta_{R}}{\varphi_{R}{}^{2}}\;0\leq y\leq 1$$

$\varphi_{L}\;and\;\varphi_{R}$ are two modified Damköhler numbers evaluated in the left and right region respectively, defined in Equations (150) and (151), $\phi^{2}$ is the Damköhler number defined in Equation (152), *y* is the dimensionless length centered in the middle plane, Equation (149) and $A_{L},A_{R},$ $B_{L},B_{R},\alpha_{L},\alpha_{R},\beta_{L},\beta_{R}$ are constants that can be found in the Supplementary Materials.

(149) y=xL2

(150) $$\varphi_{L}^{2}=\phi^{2}\left[\bar{C_{C}^{-1}}+\frac{1}{r_{C}}+\frac{1}{Kr_{AC}}\right]$$

(151) $$\varphi_{R}^{2}=\phi^{2}\left[\bar{C_{C}^{1}}+\frac{\bar{C_{A}^{1}}}{r_{C}}+\frac{1}{Kr_{AC}}\right]$$

(152) $$\phi^{2}=\frac{k_{f}L^{2}C_{A}^{-L/2}}{D_{A}}$$

In the above equations, K ins the dimensionless equilibrium constant at the upstream boundary (KCA−L2), $r_{C}$ and $r_{AC}$ are diffusivities ratio carrier/solute and complex/solute respectively. All the concentrations appearing are dimensionless and relative at the upstream solute concentration. $\bar{J_{A}}$ is the dimensionless solute flux of permeate at the boundaries *y* = −1 or *y* = 1, defined as:

$$\bar{J_{A}}=\frac{d\bar{C_{A}}}{dy}\;y=\pm 1$$

The other equations needed to close the system are the conservation of the total carrier concentration, now expressed by Equation (153), and the two integration constants, $T_{C}$ and $T_{AC}$, coming from the solution to the differential system of mass balance equations:

(153) $$\begin{array}{cc} \bar{C_{T}}=\frac{1}{2}(\frac{1}{r_{C}}- & \frac{1}{r_{AC}})\{[\frac{A_{L}}{\varphi_{L}}\left(1-\frac{1}{cosh\left(\varphi_{L}\right)}\right)+\left(1-\frac{\alpha_{L}}{\varphi_{L}{}^{2}}+\frac{\beta_{L}}{\varphi_{L}{}^{2}}\right)\frac{tanh\left(\varphi_{L}\right)}{\varphi_{L}}\\ & +\frac{\alpha_{L}}{2\varphi_{L}{}^{2}}-\frac{\beta_{L}}{\varphi_{L}{}^{2}}]\\ & -[\frac{A_{R}}{\varphi_{R}}\left(1-\frac{1}{cosh\left(\varphi_{R}\right)}\right)-\left(\bar{C_{A}^{1}}+\frac{\alpha_{R}}{\varphi_{R}{}^{2}}+\frac{\beta_{R}}{\varphi_{R}{}^{2}}\right)\frac{tanh\left(\varphi_{R}\right)}{\varphi_{R}}\\ & +\frac{\alpha_{R}}{2\varphi_{R}{}^{2}}-\frac{\beta_{R}}{\varphi_{R}{}^{2}}]\}-\frac{T_{C}}{r_{C}}+\frac{T_{AC}}{r_{AC}} \end{array}$$

(154) $$T_{C}=1-\bar{J_{A}}+r_{C}\bar{C_{C}^{-1}}=\bar{C_{A}^{1}}+\bar{J_{A}}+r_{C}\bar{C_{C}^{1}}$$

(155) $$T_{AC}=1-\bar{J_{A}}+r_{AC}\bar{C_{AC}^{-1}}=\bar{C_{A}^{1}}+\bar{J_{A}}+r_{AC}\bar{C_{AC}^{1}}$$

Together with the two new boundary conditions in *y* = 0, reported in Equation (156):

(156) $$\frac{d\bar{C_{A,L}}}{dy}=\frac{d\bar{C_{A,R}}}{dy}\;y=0\;\bar{C_{A,L}}=\bar{C_{A,R}}$$

Concentration profiles and solute flux can be calculated by solving the set of nonlinear equations derived.

Once the solute concentration profile is known, it is possible to evaluate its derivative required for the facilitation factor calculation:

(157) $$F=1+\frac{2\bar{J_{A}}}{\bar{\bar{C_{A}^{-1}}-\bar{C_{A}^{1}}}}$$

In the above equations, the superscript −1 or 1 indicates the point at which the concentration is evaluated according to the dimensionless coordinates *y,* defined in Equation (149).

In terms of facilitation factor, the results of this approximate method showed excellent agreement with the numerical solution given by Basaran [8] and Kutchai et al. [53] for different Damköhler numbers, different diffusivities ratio and downstream solute concentrations. The method is also able to predict the presence of an optimal value of equilibrium constant like the one of Teramoto [74].

**Figure 8:**
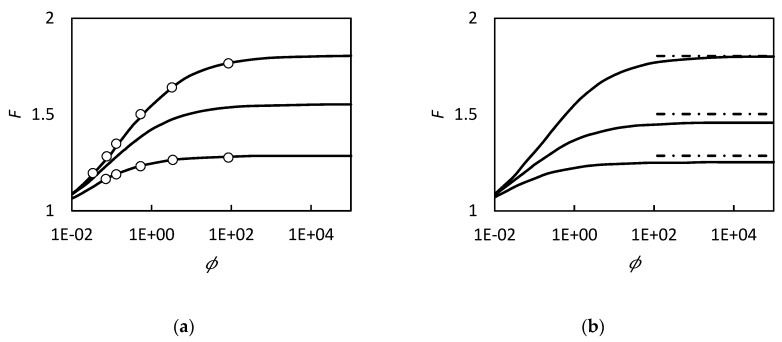
Facilitation factor as function of Damköhler number (Equation (157)). (**a**) Influence of the downstream solute concentration (dimensionless) for the case *D_AC_* = *D_C_*. From the top, $\bar{C_{A}^{1}}$ = 0, 0.1, 0.4. Circles are numerical solution from Kutchai et al. [53], lines are calculations from the present model; (**b**) diffusivities ratio effect, *r* = *D_AC_*/*D_C_*_,_ for zero downstream concentration. From the top, *r* = 1, *r* = 0.5, and *r* = 0.25. Continuous lines are the model of Morales and Cabrera [98], dash and dotted lines are the asymptotic solution of Teramoto [74].

Figure 8a shows how the facilitation factor is affected by the Damköhler number, defined in Equation (152), for different values of downstream solute concentration. The drop in facilitation factor due to the increase of backpressure is well described. In Figure 8b, it is also reported the same dependence of the facilitated factor to the ratio of carrier and carrier complex diffusivity. Moreover, the present approach can describe the equilibrium regime which occurs for the entire range of Damköhler numbers, in accordance with both the analytical equilibrium solution reported by Ward [54] and the Kutchai’s numerical solution [53].

Even if this approach represents an improvement of the one proposed by Teramoto, it suffers in terms of concentration profiles description in the middle of the membrane, for values of a Damköhler number larger than one, in the near chemical equilibrium regime [99]. In this regime, differences between the approximate and exact solutions still remain in the middle region while, near the boundaries, the solutions agree. However, looking at the overall membrane properties, in terms of solute flux or facilitation factor, similarly to Teramoto [74], the present approach can describe properly the actual behavior of these kind of systems, despite the solution procedure is not straightforward.

## 3. Fixed Carrier Systems

In this family of facilitated transport systems, the carrier agent is attached to the polymer backbone. Unlike the MCS case, the carrier cannot diffuse across the membrane but, rather, can vibrate around its equilibrium position in the chain [100]. As a general consequence of that, FSC systems are characterized by lower solute fluxes than MC ones but, on the other hand, they own a better long term stability, since the carrier agent cannot leave the membrane [48,82,101,102]. This advantage make the fixed carrier systems a valid alternative to the more common mobile carrier systems.

### 3.1. Models for Fixed Sites Facilitated Transport Membranes

The present section will start with introduction of the dual mode (DM) transport model. This model was introduced, at the end of the 1950s, to describe the gas solubility and diffusion in glassy polymers, without any explicit chemical reaction between the polymer and solute. However, since the Langmuir sorption mechanism [103], which provides one of the terms of the DM model, could be explained by chemical reaction mechanisms [104], some authors used the DM approach for the mathematical description of FSC systems.

In particular, this was done in the first studies concerning the facilitated transport of oxygen in polymer-based metal complex membranes, as in the works by Okahata et al. [24], Nishide et al. [25,105], but also to represent the selective permeation of CO_2_ in polymeric membranes having aminoethoxycarbonyl moieties (Yoshikawa et al. [106,107]). Moreover, the dual mode comprehensive transport framework derived by Barrer [108] in 1984, was used by Noble [109,110,111] to derive some crucial results about the fixed carrier facilitated transport. Even if the dual mode has been applied to many fixed carrier systems, with good results in terms of fitting, this theory does not provide a clear and full explanation of the transport mechanism in these facilitated membranes, in particular regarding the chemical reaction effect.

Cussler et al., [100], and Noble, [109,110,111], derived phenomenological models to answer that issue.

Their models, though conceptually and mathematically quite different, consider explicitly how the chemical reaction mechanism and the morphological polymer structure act on the facilitation. Cussler found that the facilitation is possible only if the carrier concentration exceeds a percolation limit, while in the works of Noble there is no such limit. One of the most interesting results of Noble’s group is that, in the condition of excess carrier concentration, the facilitation factor in FSC systems obeys the same law valid for the mobile carrier case. However, even if the work suggests a useful qualitative interpretation of the facilitation, on a quantitative point of view it works well only if the system is far from the carrier saturation condition.

Kang et al. [112,113] used a physical analogy with the parallel RC alternate voltage circuits to calculate the facilitation factor in fixed carrier membranes. In their picture, the solute transport mechanism could be seen as the electron transport in RC circuit: the pure physical diffusion is the analogous of the electron transport in the resistor element, while the reaction sites are compared to the capacitor element. A single RC circuit and a series of RC circuits was taken into account and compared.

Recently, two models have been developed by Zarca et al. [18,114], in 2017. The first one regards the pure fixed sites transport and proposes a simple mathematical relation for the permeability calculation by the use of two fitting parameters and it allows to predict the percolation concentration limit, if it exists. The second is an extension developed to describe hybrid facilitated systems in which both mobile and fixed carriers act simultaneously.

#### 3.1.1. Dual Mode Theory

The dual mode transport theory indicates a family of transport models developed by different authors. The fundamentals of such theory were derived between the late 1950s and the mid 1980s, originally to explain the excess of gas sorption in glassy polymer with respect to the value predicted by Henry’s law [115], valid for the rubbery systems. It was Meares, in 1954, [116] who hypothesized that a population of microholes exists in a glassy polymer matrix, which could contain the excess of sorbed molecules. Barrer, in 1958, firstly proposed the mathematical formulation of the gas solubility in those matrices [117]. He observed that the equilibrium concentration of solutes in glassy polymers could be described as sum of two different contribution: the Henry’s law [115] to consider the gas dissolved in the polymer matrix, and a Langmuir [103] term to take into account the gas adsorbed in the holes. The sum of these two contributions gives Equation (158), which is the dual mode sorption relation:

(158) $$C_{A}=C_{D}+C_{H}=k_{d}p+\frac{CH^{\prime }bp}{1+bp}$$

where:

$C_{A}$ = solute concentration in polymer phase
$C_{H}$ = solute concentration trapped in ‘holes’
$C_{D}$ = solute concentration dissolved
p = solute partial pressure
$k_{d}$ = Henry’s constant
$CH^{\prime }$ = holes saturation level concentration
b = affinity constant

Equation (158) is used in all the subsequent developments of the dual mode sorption theory to represent gas solubility. Some variations were proposed by considering the fugacity or activity rather than the pressure of the gas phase, as done for example by Petropoulos [118], but on the conceptual point of view nothing changes. For simplicity’s sake, we report all the model equations in terms of concentration and pressure.

The representation of gas diffusion in such systems followed a very different destiny, and many approaches were proposed in the literature, which are recalled hereafter.

In 1963 Michaels and Vieth [119], while studying gas diffusion in poly-ethylene terephthalate, considered that the diffusion coefficient of molecules trapped in the polymer “holes” should be zero, so that the gas sorbed by Langmuir mechanism is totally immobilized in the matrix. Moreover they considered that, in every point of the polymer, there is local chemical equilibrium between dissolved and adsorbed gas.

In this case, it is possible to express the adsorbed concentration as function of the dissolved one, through Equation (159):

(159) $$C_{H}=\frac{CH^{\prime }MC_{D}}{1+MC_{D}}$$

where $M=b/k_{d}$.

Based on the previous hypothesis, the solute permeability should be given simply by the product between Henry’s constant and the diffusion coefficient of dissolved species, Equation (160).

(160) $$P=k_{d}D_{d}$$

Petropoulos in 1970 [118] pointed out that some assumptions previously used to treat the diffusion in glassy polymers were not based on theoretical justifications. He focused mainly on removing the total immobilization hypothesis for adsorbed species, providing an equation to account for the diffusivity of those molecules.

Following that idea, it has been shown that the solute overall permeability has the following form:

(161) $$P=k_{d}D_{d}+\frac{CH^{\prime }D_{h}}{\left(p_{A}^{0}-p_{A}^{L}\right)}ln\frac{\left(1+bp_{A}^{0}\right)}{\left(1+bp_{A}^{L}\right)}$$

where $D_{d},D_{h}$ indicates the diffusivity of dissolved and adsorbed species respectively, $p_{A}^{0},p_{A}^{L}$ are the upstream and downstream solute partial pressures. Equation (161) still holds even if the local chemical equilibrium condition is not considered.

In 1976 Paul and Koros, [120], and Koros et al. [121] provided a mathematical description of transport in glassy polymers, to take into account the partial immobilization of the adsorbed solute. They assumed that a fraction of adsorbed molecules, together with the totality of dissolved ones, can freely diffuse in the polymer matrix, while the remaining part of the Langmuir’s type species is frozen in the holes with zero mobility.

This leads to rewrite the Fick’s law, Equation (10), for the flux as:

(162) $$J_{A}=-D\frac{dC_{m}}{dx}$$

where $C_{m}$ is the concentration of solute able to diffuse, and is given by the following equation:

(163) $$C_{m}=C_{D}\left(1+RC_{H}\right)$$

The ratio between the diffusivity coefficients of adsorbed and dissolved molecules, $D_{h}$ and $D_{d}$, indicated with *R* in Equation (163), is the measure of the partial immobilization of the Langmuir’s type species. Neglecting the downstream partial solute pressure, by means of Equations (162) and (163) one finds the relation for the permeability in the form:

(164) $$P=k_{d}D\left(1+\frac{RCH^{\prime }b}{k_{d}\left(1+bp_{A}^{0}\right)}\right)$$

Extending the idea of incomplete immobilization, Barrer in 1984 investigated the different diffusion pathways allowable in such systems and how they affect the overall transport in glassy polymers [108]. Four diffusion steps were hypothesized to occur and analyzed in detail. For each of these steps, a corresponding diffusive flux is generated:

(165) $$D_{dd}=>\;J_{dd}=-D_{dd}\frac{dC_{D}}{dx}\;\mathrm{flux}\;\mathrm{within}\;\mathrm{the}\;\mathrm{dissolved}\;\mathrm{phase}$$

(166) $$D_{dh}=>\;J_{dh}=-D_{dh}\left[\frac{dC_{D}}{dx}\left(1-\theta \right)+\frac{C_{D}}{CH^{\prime }}\frac{dC_{H}}{dx}\right]\;\mathrm{flux}\;\mathrm{from}\;\mathrm{the}\;\mathrm{dissolved}\;\mathrm{to}\;\mathrm{the}\;\mathrm{adsorbed}\;\mathrm{phase}$$

(167) $$D_{hd}=>\;J_{hd}=-D_{hd}\frac{dC_{H}}{dx}\;\mathrm{flux}\;\mathrm{from}\;\mathrm{the}\;\mathrm{adsorbed}\;\mathrm{to}\;\mathrm{the}\;\mathrm{dissolved}\;\mathrm{phase}$$

(168) $$D_{hh}=>\;J_{hh}=-D_{hh}\frac{dC_{H}}{dx}\;\mathrm{flux}\;\mathrm{within}\;\mathrm{the}\;\mathrm{adsorbed}\;\mathrm{phase}$$

where θ in Equation (166) is the ratio between the holes adsorbed concentration, $C_{H}$ and the saturation holes concentration, $CH^{\prime }$:

(169) $$\theta =\frac{C_{H}}{CH^{\prime }}$$

The sum of the four parallel contributions, written as function of dual mode concentration gradients in Equations (165)–(168), gives the solute total flux:

(170) $$J=J_{dd}+J_{hd}+J_{dh}+J_{hh}=-\left[D_{dd}+D_{hd}\left(1-\theta \right)\right]\frac{dC_{D}}{dx}-\left[D_{hh}+D_{hd}+D_{dh}\frac{C_{D}}{CH^{\prime }}\right]\frac{dC_{H}}{dx}$$

Barrer also showed that the overall solute diffusivity may be expressed as:

(171) $$D=\left\{D_{dd}\left(C_{D}^{0}-C_{D}^{L}\right)+\left(D_{hd}+D_{hh}\right)\left(C_{H}^{0}-C_{H}^{L}\right)-\frac{D_{dh}}{M}\left[\left(\theta_{0}-\theta_{L}\right)+2ln\frac{\left(1-\theta_{0}\right)}{\left(1-\theta_{L}\right)}\right]\right\}\frac{1}{\left(C_{A}^{0}-C_{A}^{L}\right)}$$

where D is the overall solute diffusivity, and superscripts 0, *L* indicate the upstream and downstream side, respectively. If the downstream solute pressure goes to zero, $C_{A}^{L}\ll C_{A}^{0}$, Equation (171) becomes:

(172) $$D=\left\{D_{dd}C_{D}^{0}+\left(D_{hd}+D_{hh}\right)C_{H}^{0}-\frac{D_{dh}}{M}\left[\theta_{0}+2ln\left(1-\theta_{0}\right)\right]\right\}\frac{1}{C_{A}^{0}}$$

Equation (172) allows to write the following expression for the overall solute permeability:

(173) $$P=k_{d}\left\{D_{dd}+\left[\left(D_{hd}+D_{hh}\right)KCH^{\prime }-D_{dh}\right]\left(1-\theta_{0}\right)-2D_{dh}\frac{\left(1-\theta_{0}\right)}{\theta_{0}}ln\left(1-\theta_{0}\right)\right\}$$

From Equation (173) we can obtain useful information about two limiting cases. For sufficiently low pressure values, the Langmuir saturation ratio, θ, goes to zero indicating that the holes are nearly free. In this case, Equation (173) reduces to:

(174) $$P=k_{d}\left\{D_{dd}+\left[\left(D_{hd}+D_{hh}\right)MCH^{\prime }+D_{dh}\right]\right\}$$

At the opposite, the other limiting case arises for sufficiently high pressure values. In such case the Langmuir sites saturation occurs, θ goes to one and the permeability approaches the following value:

(175) $$P=k_{d}D_{dd}$$

Note that the latter equation states that in the saturated condition, the permeability is related only to the mobility of the dissolved phase, as in the case of Equation (160).

In Equation (176) below, we reported the ratio between Equations (174) and (175). As we can see, that ratio is always higher than or equal to one, indicating that in these systems, for which the adsorbed species are partly free to move across the film, the permeability decreases as the holes undergo saturation.

(176) $$\beta =k_{d}\left\{1+\frac{\left[\left(D_{hd}+D_{hh}\right)MCH^{\prime }+D_{dh}\right]}{D_{dd}}\right\}$$

The interpretation provided by Barrer in 1984 represent the most complete description of the possible mechanism of gas transport in gassy polymer systems offered by the dual mode theory. The previous key results, Equations (165)–(168) and (170)–(176), were also confirmed one year later, in 1985, by Fredrickson and Helfand [122], who were not aware of Barrer’s published results. Moreover, they demonstrated that the same relations could be derived starting from a microscopic scale, using a lattice matrix structure, and a mathematical treatment based on probability distribution functions. Different authors, investigating the facilitated transport by fixed carriers, explained and modeled the experimental data using the dual mode as a true physical theory. It is easy to see that the Langmuir sorption model, reported in Equation (158), is the equivalent of Equation (24) reported in the mathematical formulation section.

**Figure 9:**
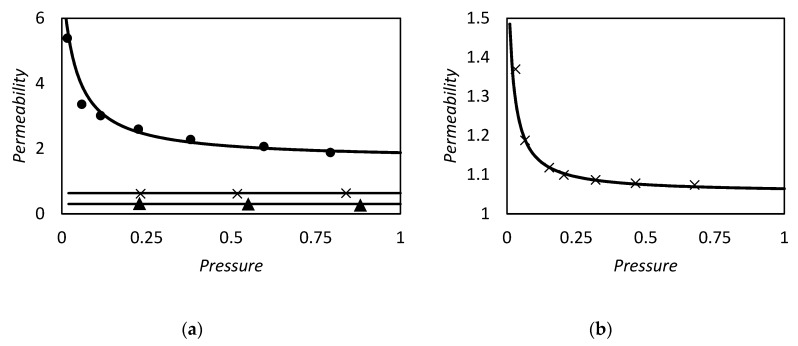
Dual mode model results, permeability as function of upstream pressure. (**a**) Yoshikawa et al. [106]. Circles, crosses and triangles are CO_2_, O_2_ and N_2_ measured, respectively; (**b**) Nishide et al. [105]. Lines are the model predictions. Permeability is in (1 × 10^−9^ × cm^3^·cm/(cm^2^·s·atm)), pressure is in (atm).

To the best of our knowledge, Okahata et al. [24] were the first ones to test the dual mode model on FSC systems for oxygen/nitrogen separation membranes in 1986. They synthesized a fixed sites carrier membrane by dispersing, in poly-butyl methacrylate, a cobalt-porphyrin-imidazole complex (CoPIm) that reacts reversibly and rapidly with molecular oxygen and the permeability results were modeled with the Paul and Koros interpretation of the dual mode [120]. Yoshikawa et al. [106], in 1988 investigated the selective permeation of carbon dioxide, over nitrogen and oxygen, in poly(4-vinylpyridine-co-acrilonitrile) membranes. They found that, while for the inert gases, O_2_ and N_2_, the permeability was independent from the upstream pressure, for the carbon dioxide the behavior was quite different. Their data, shown below in Figure 9a, evidence a decrease in permeability as the upstream pressure increases, in agreement with the saturation mechanism explained. Even in this case, the experimental permeability of carbon dioxide was modeled by the partial immobilization model of Paul and Koros, Equation (164) [120].

A facilitation effect was detected by Nishide et al. [25,105] in the oxygen permeation across a polymeric membranes containing cobalt porphyrin as fixed carrier. Also in this case, oxygen permeability was found to depend on the upstream pressure. The experimental measurements were interpreted with the Barrer equation for permeability, Equation (173), [105], and with the Paul and Koros model, reported in Equation (168), in ref. [25], providing satisfactory fitting results (Figure 9b). The permeability equation derived by Paul and Koros was also used successfully by Yoshikawa et al. in 1995 [107] in the facilitated transport of carbon dioxide across pyridine moiety fixed carrier membrane.

#### 3.1.2. Cussler et al., 1989. On the Limits of Facilitated Diffusion

Cussler et al. [100], in 1989, explained the fixed carrier transport mechanism and provided an expression for the solute flux. In particular, they considered the membrane as a series assembly of contiguous lamellae, with one carrier site per lamella. Since the carrier is fixed, it cannot diffuse freely in the membrane, but it is allowed to oscillate around its equilibrium position in the matrix, due to vibrational energy. The same limitation still holds also for the reaction product. Moreover, the authors considered that the free solute does not exist in the polymer phase, so that pure physical diffusion cannot occur. Another hypothesis in the present treatment is that the solute-carrier chemical reaction, Equation (3), takes place only at the membrane surfaces while, inside the membrane, only an exchange mechanism between carrier sites is present. Hence, in some conditions, the complex could hop from one carrier site to another.

**Figure 10:**
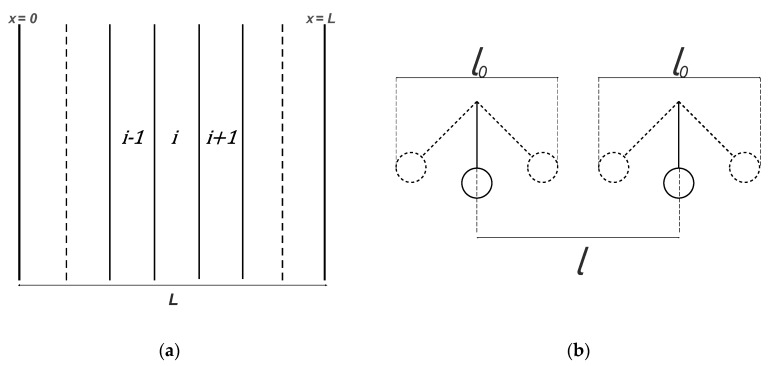
Representation of membrane thickness in Cussler et al. [100]. (**a**) Series arrangement of contiguous lamellae. *L* is the membrane thickness. (**b**) Contiguous fixed sites. *l*_0_ is the oscillation width, *l* is the distance between equilibrium positions. For *l* > *l*_0_ solute exchange between contiguous sites cannot occur.

Figure 10 reports schematically the description of transport used by the authors. The continuous line circles represent the fixed sites in equilibrium position, while the dashed ones represent the carriers located at the two ends of oscillation. Hence, l indicates the space between contiguous equilibrium positions, while *l*_0_ is the amplitude of the oscillation. If the distance between equilibrium locations is higher than the oscillation width, the carriers cannot get in touch and no solute exchange is possible. In this case, no diffusion flux can be observed. Conversely, if $l_{0}\geq l$ two carriers could get in contact and an exchange mechanism can take place. The latter condition is therefore necessary for the solute transport. If that condition is achieved, the equation for the flux is:

(177) $$J_{A}=\frac{D}{L}\left(C_{C}^{L}-C_{C}^{0}\right){\left[\left(2-\frac{l_{0}}{l}\right)+\left(\frac{l_{0}}{l}-1\right)\left(\frac{1}{2}+\frac{1+cosh\left(\psi \right)}{\psi sinh\left(\psi \right)}\right)\right]}^{-1}$$

(178) $$\psi =\sqrt{\frac{2kC_{T}{\left(l_{0}-l\right)}^{2}}{l_{0}D}}$$

where ψ, defined in Equation (178) above, is the Thiele modulus (or Damköhler number) accounting for the ratio between chemical reaction and diffusion rate. In the previous equation, the constant reaction rate, k, represents the solute exchange rate between two contiguous sites and D is not an intermolecular complex diffusivity, but rather it is the intramolecular diffusion coefficient, i.e., the diffusivity across polymer chains by the proposed jumping mechanism. If the equilibrium condition is considered, Equation (177) becomes:

(179) $$J_{A}=\frac{D}{L}\left(\frac{C_{T}l}{l_{0}}\right)\frac{K_{eq}C_{A}^{0}}{\left(1+K_{eq}C_{A}^{0}\right)}{\left[\left(2-\frac{l_{0}}{l}\right)+\left(\frac{l_{0}}{l}-1\right)\left(\frac{1}{2}+\frac{1+cosh\left(\psi \right)}{\psi sinh\left(\psi \right)}\right)\right]}^{-1}$$

where the first term in brackets represents the average total carrier concentration in every lamella.

The latter equation presented was used to obtain some important results in the two limiting case of facilitated transport.

For the fast reaction case, indeed, the Thiele modulus is large and the flux is given simply by:

(180) $$J_{A}=\frac{D}{L}\frac{C_{T}K_{eq}C_{A}^{0}}{\left(1+K_{eq}C_{A}^{0}\right)}\left(\frac{2l/l_{0}}{3-l_{0}/l}\right)$$

The same results of Equation (180) could be easy obtained for the mobile carrier case by neglecting the pure physical diffusion mechanism with the only difference of the term in brackets (see the mathematical background section). If we consider $l=l_{0}$, the difference between the two cases vanishes.

On the other hand, the most interesting case is the one for which the diffusion mechanism is faster than the reaction. In this regime ψ goes to zero, and it is possible to obtain an expression for the apparent diffusion coefficient related to both morphological and reaction parameters. In this case, the equation for the flux becomes:

(181) $$J_{A}=\frac{\left[kC_{T}l^{3}\left(l_{0}-l\right)/l_{0}^{2}\right]}{L}\frac{C_{T}K_{eq}C_{A}^{0}}{\left(1+K_{eq}C_{A}^{0}\right)}$$

Equation (181) shows the flux expression for fast diffusion regime. The apparent diffusion coefficient here is the term in square brackets and contains both characteristic lengths involved, $l_{0},\;l$, as well as the exchange solute rate, k.

**Figure 11:**
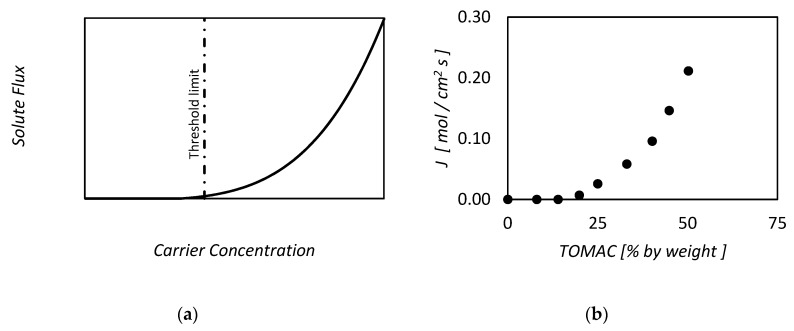
Solute flux as function of carrier concentration. (**a**) Qualitative behavior in presence of a threshold limit. For carrier concentration below the threshold, the solute flux does not occur; (**b**) experimentally detected fructose flux in plasticized cellulose triacetate as function of carrier (TOMAC) concentration, Riggs and Smith [125].

As stated, the solute exchange between adjacent sites is possible only if $l_{0}\geq l$, and this implies the presence of a percolation threshold, i.e. a carrier concentration limit below which no transport is possible (Figure 11a). In fact, while the oscillating width is strictly correlated to the polymer nature, the distance l is strongly carrier concentration dependent. The higher the concentration, the shorter the distance, and vice-versa. Although the authors did not consider the free solute diffusion, their model is able to capture the transport nature of some of these carrier systems.

As reported in many works about FSC systems [114,123,124,125,126], a low carrier concentration limit could exist and the solute flux is observed only above certain concentration values. In Figure 11b, we report the carrier-mediated flux of fructose in plasticized cellulose triacetate membrane using trioctymethilammonium chloride (TOMAC) as carrier, by Riggs and Smith [125]. However, the mechanism proposed by Cussler cannot explain cases in which the solute flux differs from zero, even at low carrier concentrations, as the facilitated transport of oxygen reported by Tsuchida et al. [127] or the transport of organic acids studied by Yoshikawa et al. [128].

#### 3.1.3. Noble, 1990. Analysis of Facilitated Transport with Fixed Site Carrier Membranes

Noble in 1990 presented the first of three papers about the FSC facilitated transport [109]. The other two papers [110,111] were published in 1991 and 1992. The works deeply investigate the nature of facilitation in these systems and provide a mathematical relation between the four diffusion coefficients, previously introduced in the dual mode framework by Barrer [108], and the chemical reaction mechanism between carrier and solute molecules.

In the case of large excess of carrier, which implies that the concentration of free carrier, *C_C_*, is nearly constant and not too different from the total carrier concentration *C_T_*, Noble found that Equation (13) describes the complex mass balance also in the fixed carrier case. The derivation of such equation requires the existence of the four exchange mechanisms introduced by Barrer, [108], conversely to what Cussler did [100].

**Figure 12:**
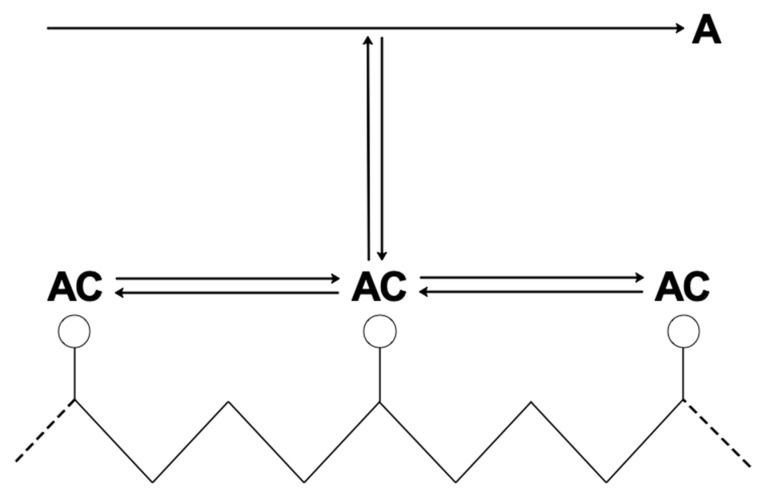
Solute transport pathways in fixed sites carrier systems used by Noble [109].

In Figure 12 the transport paths are shown. The horizontal lines at the top indicate the pure free solute physical diffusion. The double lines indicate the exchange mechanisms between adjacent fixed sites, (horizontal ones) or between a fixed site and the free solute region (vertical ones).

Even if the Equation (13) still holds in this case, the diffusion coefficient appearing, *D_AC_*, is now function of the morphology of the matrix and of the mobility between sites. That function was found to be in the form:

(182) $$D_{AC}=l^{2}\bar{k}$$

where l is the distance between two fixed sites and $\bar{k}$ is a mobility parameter.

For zero downstream pressure, with the assumption of excess carrier and since the mathematical transport problem for solute and reaction product is identical to that in liquid membranes, the solution for the facilitation factor is equivalent to the one of Smith and Quinn [93], already discussed in the previous section and hereafter shown:

$$F=\frac{\left(1+\frac{\alpha K}{1+K}\right)}{1+\frac{\alpha K}{1+K}\frac{tanh\left(\lambda \right)}{\lambda }}$$

The parameters reported in Equation (116) have been already explained in previous section; the “only” difference between the present and the mobile carrier case is the physical meaning of the complex diffusivity, here given by Equation (182) but not fully explained. In particular, the relation between the complex diffusivity and the chemical reaction was not given in the present work and presented only later, in the subsequent works of 1991 and 1992.

If the chemical equilibrium is taken into account, tanh(λ)/λ goes to zero and the facilitation factor is simply given by:

(183) $$F=\left(1+\frac{\alpha K}{1+K}\right)$$

This result, valid in the chemical equilibrium regime, is analogous to the one achievable by the dual mode theory and was tested to describe the experimental data of Tsuchida et al. [127] concerning the facilitated transport of molecular oxygen in a polymer with cobalt Schiff complex bases as fixed carrier.

**Figure 13:**
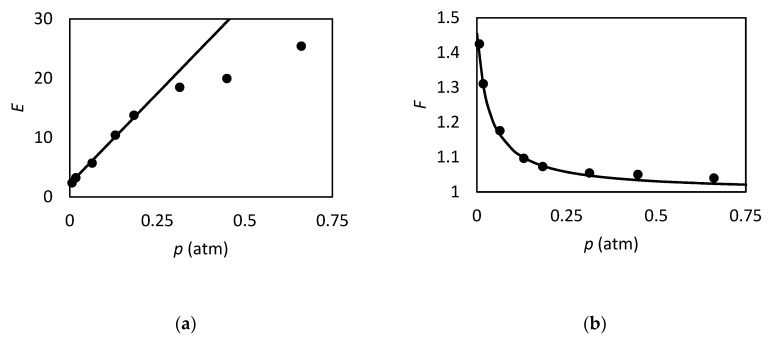
Modeling results using Equation (3.22). (**a**) Plot of *E* = (*F* − 1)^−1^ as function of upstream pressure. At high pressure, the linearity is lost. (**b**) Facilitation factor calculated by using parameters retrieved in the low pressure range of figure a. Circles are experimental data from Tsuchida et al. [127], lines come from Equation (183).

By plotting $E={\left(F-1\right)}^{-1}$ against feed side gas pressure, it is possible to retrieve the parameters needed to model the system [129,130,131]. Figure 13 reports the results obtained by using this approach: as shown in Figure 13a, after a certain value of upstream pressure linearity dependences is lost and, after that, the solution given in Equation (183) is no longer valid. However, the use of the latter equation in its range of applicability could be sufficient to retrieve the parameters values required to describe the system in all the range of interest, as shown in Figure 13b. With simple algebra it is possible to demonstrate that the intercept of the plot given in Figure 13a is ${\left(\alpha K\right)}^{-1}$ while the slope is $\alpha^{-1}$.

In his work in 1990, Noble obtained two key results which he then used in the following studies. They were:

- the mathematical derivation of the mass balance analog of the mobile carrier case, which allows to use, in analytical approximation methods already known, Equation (2.89) while an excess of carrier is considered.
- the functional dependence of the actual complex diffusivity in such systems on morphological and chemical parameters, Equation (182).

#### 3.1.4. Noble, 1991, Facilitated Transport Mechanism in Fixed Site Carrier Membranes

In 1991 Noble extended his previous work and, starting from Equation (183), derived a series of equivalences between the Barrer’s diffusion coefficients [108] and the equilibrium reaction constant in the excess carrier condition and diffusion limited regime [110]. Moreover, he found that the facilitation could be achieved, theoretically, even if the direct diffusion between carrier sites, *D_hh_* in Barrer notation Equation (168), is equal to zero.

To derive the proper set of equivalences, he started from the equation for the total solute flux, Equation (170). The latter can be rewritten in terms of carrier, free solute and complex concentrations as:

(184) $$J=J_{dd}+J_{hd}+J_{dh}+J_{hh}=-\left[D_{dd}+D_{hd}\left(\frac{C_{C}}{C_{T}}\right)\right]\frac{dC_{A}}{dx}-\left[D_{hh}+D_{hd}+D_{dh}\left(\frac{C_{A}}{C_{T}}\right)\right]\frac{dC_{AC}}{dx}$$

The above equation ensures that facilitation is allowable even if $D_{hh}=0$, as in the case of low carrier concentration (i.e., when the sites are too far apart and the hopping mechanism cannot occur). Indeed, if Equation (184) holds, the condition for pure physical diffusion is achieved when the carrier is saturated, i.e., when $C_{C}\to 0$ and also $\frac{dC_{AC}}{dx}\to 0$. For this reason, thanks to the additional transport pathways considered, no percolation threshold appears a priori even for $D_{hh}=0$.

In the chemical equilibrium regime, the complex gradient could be related to the free solute gradient by Equation (159). As a result, the solute gradient induces a complex gradient which enhances the transport up to the saturation. The same result may be casted in the facilitation factor terms that, even by neglecting the direct site-site exchange, $D_{hh}=0,$ the facilitation factor is not equal to one, but has the following form:

(185) $$F=1+\frac{D_{dh}C_{C}}{D_{dd}C_{T}}+\frac{D_{hd}}{D_{dd}}\left(\frac{KC_{T}}{C_{A}^{0}}\right)\frac{C_{C}}{C_{T}}$$

Equation (185) again shows that facilitation occurs up to the carrier saturation, i.e., when free carrier sites are no longer present, $C_{C}=0$.

The relationship between the actual complex diffusivity and the exchange mechanism between the free diffusion path and the fixed sites region was found by equating Equations (185) and (183) and is given by:

(186) $$D_{AC}=D_{hd}+D_{dh}\left(\frac{C_{A}^{0}}{KC_{T}}\right)$$

The above equation is a crucial point in the analysis provided by Noble in 1991. He demonstrated in a rigorous way, even if in the excess carrier case, that the effective complex diffusivity depends from the chemical reaction, by dimensionless chemical equilibrium K, and from the morphological structure of the matrix, strictly affected by the total carrier concentration, $C_{T}$. Note that, since K is the dimensionless reaction equilibrium constant, $K_{eq}C_{A}^{0}$, the complex diffusivity, $D_{AC}$, does not depend on the upstream concentration.

Moreover, also the two diffusion coefficients $D_{hd}$ and $D_{dh}$ could be correlated to the chemical reaction, responsible of the complex formation and consumption.

For the low pressure range, Noble found that the ratio between the two diffusion coefficients is related to dimensionless constant and to the total carrier concentration through a simple mathematical relationship which correlates the two phenomena:

(187) $$K=\frac{D_{dh}C_{A}^{0}}{D_{hd}C_{T}}$$

In chemical equilibrium regime, therefore, the exchange diffusion mechanism acting between fixed sites and free diffusional path is related to the chemical equilibrium constant. In other words, the higher the forward kinetic constant, $k_{f},$ the higher the exchange of solute from the physical diffusion region to the fixed site.

Equation (187) can be rearranged and inserted in Equation (186) to provide the final relation, Equation (188), that allows, together with Equation (3.26), to determine $D_{dh}$ and $D_{hd}$ once $D_{AC}$ is known.

(188) $$D_{AC}=2D_{hd}$$

The main result of the work of 1991 is the demonstration that, theoretically, facilitation can occur even if $D_{hh}=0$, so that the direct jumping mechanism is not the only one influencing the carrier-mediated transport. The complex diffusivity in such systems is related to chemical reaction parameters and morphological, carrier concentration dependent, parameters by Equations (186), (187) and (188). That solution will be improved by Noble in a subsequent, and more detailed, work [111], hereafter reported, where the final equation that combines all the variables influencing the complex mobility is given.

#### 3.1.5. Noble, 1992, Generalized Microscopic Mechanism of Facilitated Transport in Fixed Site Carrier Membranes

In 1992 [111] Noble provided his general theory for the facilitated transport across FSC membranes. The direct hopping mechanism between fixed sites was included in this final description. He found a general equation for the effective complex diffusivity, $D_{AC}$, that accounts for the morphological nature of the systems and for the chemical complexing reaction between solute and carrier sites. The dual mode equations by Barrer were used, again, for the total flux, adapted in Equation (184).

As stated above, when two sites are far apart it is not possible for the solute to jump from one site to another. From a macroscopic point of view, considering a homogeneous distribution of carrier sites, this happens for low carrier concentrations. Cussler concluded that in this case no facilitation can occur, [100], while previously Noble had found that the facilitation is still allowable due to the presence of the exchange mechanism related to the complexation reaction [109].

In general, the flux of solute across these systems is function of both free solute and complex gradients across the membrane boundaries by Equation (184). However, as carrier concentration becomes small, in certain zones, Equation (184) reduces simply to:

(189) $$J=J_{dd}=-D_{dd}\frac{dC_{A}}{dx}$$

because carrier is no longer present locally to react with the solute. However, near the sites, Equation (184) still holds.

**Figure 14:**
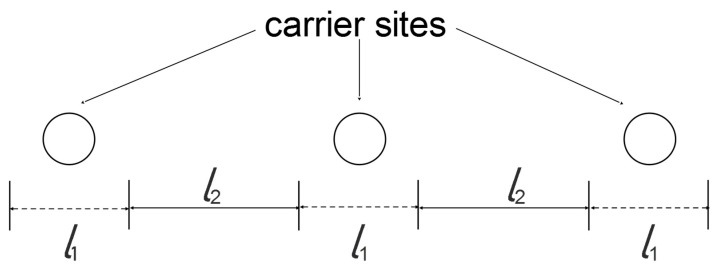
Membrane environment in the present work of Noble. Between sites, in the length space *l*_2_, pure diffusion mechanism exists. Closely to the sites position, *l*_1_, Equation (184) holds.

In Figure 14 we report the internal structure of the membrane used by Noble in 1992. Similar to Figure 10, the circles are the fixed carriers, while $l_{1}$ and $l_{2}$ are the two length scales. The first one represents the space of influence of the carrier, in other words the space around a fixed site for which Equation (184) describes the local solute flux. The second, $l_{2}$, measures the space between carriers in which the solute transport follows Equation (189).

By taking in account these two lengths, the facilitation factor was derived as:

(190) $$F=\frac{A+\beta B}{D_{dd}f+\left(A+\beta B\right)\left(1-f\right)}$$

where f is the length ratio defined as:

(191) $$f=\frac{l_{1}}{l_{1}+l_{2}}$$

and the other symbols appearing in Equation (190) are defined below:

(192) $$\beta =\frac{K_{eq}C_{T}}{{\left(1+K_{eq}C_{A}\right)}^{2}}$$

(193) $$A=D_{dd}+D_{dh}\frac{C_{C}}{C_{T}}$$

(194) $$B=D_{hh}+D_{hd}+D_{dh}\frac{C_{A}}{C_{T}}$$

Assuming the chemical equilibrium and considering the case of low upstream pressure, the facilitation factor reduces to:

(195) $$F=1+\beta \frac{D_{AC}}{D_{dd}}$$

which can be equated to Equation (190), to obtain the complex diffusion coefficient.

(196) $$D_{AC}=\frac{1}{\beta }\left[\frac{A+\beta B-D_{dd}}{1+\left(\frac{A+\beta B}{D_{dd}}\right)\left(\frac{1-f}{f}\right)}\right]$$

The resulting equation, Equation (196), shows how the actual diffusion coefficient of the complexed species is affected by morphological and chemical reaction effects.

From the above results, it is possible to analyze two opposite cases. First, if the sites are too distant for a direct hopping mechanism, the diffusion coefficient related to that, $D_{hh}$, is obviously zero and the complex diffusivity can be rewritten in the following form:

(197) $$D_{AC}=D_{dd}\left(\frac{f}{1-f}\right)\left(\frac{R}{D_{dd}+\beta R}\right)$$

where R is a parameter containing the reaction contribution to the transport and defined as:

(198) $$R=D_{hd}+D_{dh}\left(\frac{C_{A}}{C_{T}}+\frac{C_{T}-C_{AC}}{K_{eq}C_{T}{}^{2}}\right)$$

On the contrary, if the sites are adjacent, the direct jump of solute from one carrier site to the next one is possible and $D_{hh}\neq 0$. This happens if the length $l_{2}$ approaches zero; the complex diffusivity coefficients is then given by:

(199) $$D_{AC}=D_{hh}+R$$

As we can see, the pure physical diffusion in this case does not influence the complex transport as in Equation (199), $D_{dd}$ does not appear.

These two latter results, Equations (197)–(199), complete the description of Noble about the facilitation in FSC membranes that started three years earlier, in 1990 [109]. The analysis provided by the author is based on some approximations and simplifications, excess of carrier, chemical equilibrium, dilute solute concentration, but still provides an interesting picture of transport in these systems.

In conclusion, in the Generalized Microscopic Mechanism of Facilitated Transport in Fixed Site Carrier Membranes, the general mathematical formulation of the complex diffusion coefficient was derived in terms of morphological and reaction parameters. It was demonstrated theoretically that facilitation can occur even if the carrier sites are too far and the solute cannot hop directly from one to another.

#### 3.1.6. Kang et al., 1995. Analysis of Facilitated Transport in Solid Membranes with Fixed Site Carriers

An interesting theoretical model for the interpretation of facilitated transport in FSC membranes was given in 1996 by Kang et al. [112,113]. Their work, divided in two papers, studies the permeability in these systems by means of analogies with resistor–capacitor circuits (RC), in parallel arrangement, in alternating voltage regime. In the first paper they reported the single circuit case, [112], while in the second one, [113], the solution was extended to the case of a series RC circuit arrangement. Here we present the general solution given for a series circuits, since the single circuit case is a particular solution of that.

In both RC circuits and in FSC membranes, the transport of particles occurs due to a driving force imposed at the boundaries of the system. For the electrical circuit case, the particles are electrons and the driving force is the voltage gradient; for the FSC case, the flux of solute molecules is related to a concentration gradient. Moreover, in the latter case the solute flows through different pathways, as already mentioned by Noble [111]. Even if a parallel RC circuit is considered, the electrons flow separately in two distinct paths and, for the Kirchoff’s rules, the total electron flux is equal to the sum of the two single contributions, Equation (200)

(200) $$j_{e}=j_{r}+j_{c}$$

where $j_{e}$ is the total electrons flux and $j_{r},j_{c}$ are the resistor and capacitor contributions respectively.

Furthermore, as in the case of carrier saturation, for which the facilitation effect disappears, when the charge on the condenser element reaches its maximum value, also the second term in the right hand side of Equation (200) vanishes, leaving only the resistance contribution.

**Figure 15:**
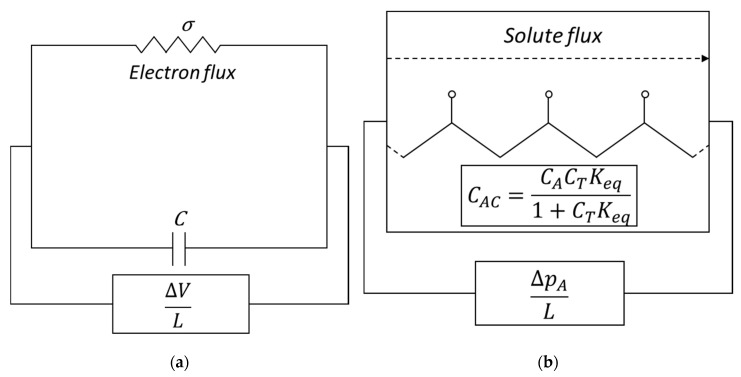
Analogies between RC parallel circuit and fixed sites carrier systems. (**a**) Parallel configuration of single RC circuit; (**b**) fixed sites carrier systems. Analogies among driving forces and capacitance effects according to Table 1.

With the above considerations, the authors concluded that the pure physical diffusion could be compared to the resistor element and that the fixed sites behave similarly to the condenser in the RC parallel configuration. In Figure 15, below, we report the schematic pictures of a general RC circuit and a FSC systems. The general relationship between voltage driving force and resistor electron flux is expressed by the Ohm’s law:

(201) $$j_{e}=\sigma \frac{\Delta V}{L}$$

where σ is the conductivity, ΔV is the voltage difference, L is the length of the system.

By comparing the previous equation with the Fick’s law written in terms of permeability, for zero downstream pressure, the following analogies could be revealed:

As shown in Table 1, the complex concentration is the one in chemical equilibrium with carrier and solute.

The alternating voltage regime of RC circuit is then replaced by a fluctuating local concentration. That fluctuation is attributed to the presence of chemical equilibrium reaction: the forward reaction lowers the solute concentration, while the inverse reaction has the opposite effect.

As a result, the solute concentration value is the sum of two different terms: the first equals the equilibrium value and the other is expressed as sinusoidal function of time, related to the direct and inverse reaction rate. If we consider a system composed by a series of *n*-*RC* parallel circuits, the following solution for the electrical conductivity can be derived:

(202) $$\frac{\sigma_{RC}}{\sigma }=1+\alpha_{V}\sqrt{n^{2}+{\left(\frac{C\omega L}{A_{s}\sigma }\right)}^{2}}$$

where $\alpha_{V}$ is the extent of voltage fluctuation, ω is the frequency, $A_{s}$ is the flux surface, C is the capacitance, $\sigma_{RC}$ is the total conductivity, σ is the resistance conductivity and *n* is the number of circuits composing the series.

By using the proper substitution, the solution for the facilitation factor, expressed as ratio between permeability of facilitated systems divided by the pure physical diffusion contribution, is reported in Equation (203)

(203) $$F=1+\alpha_{p}\sqrt{n^{2}+{\left[2\pi \psi \gamma ln\left(1+K\right)\right]}^{2}}$$

where:

$$\alpha_{p}=\frac{C_{A}^{d}}{C_{A}^{0}}$$

$$\psi =\frac{k_{r}L^{2}}{D_{A}}$$

$$\gamma =\frac{C_{T}}{C_{A}^{0}}$$

$$K=K_{eq}C_{A}^{0}$$

The parameter n in Equation (203) is the number of RC circuits in the series. By setting *n* = 1 the single case is obtained [112].

The extent of concentration fluctuation, $\alpha_{p}$, is essentially a fitting parameter since $C_{A}^{d}$ represents the perturbation amplitude, that does not have a clear physical meaning. Moreover, the concentration fluctuations, if existing, probably have a more complicated mathematical form than the one used by the authors as sinusoidal function.

**Figure 16:**
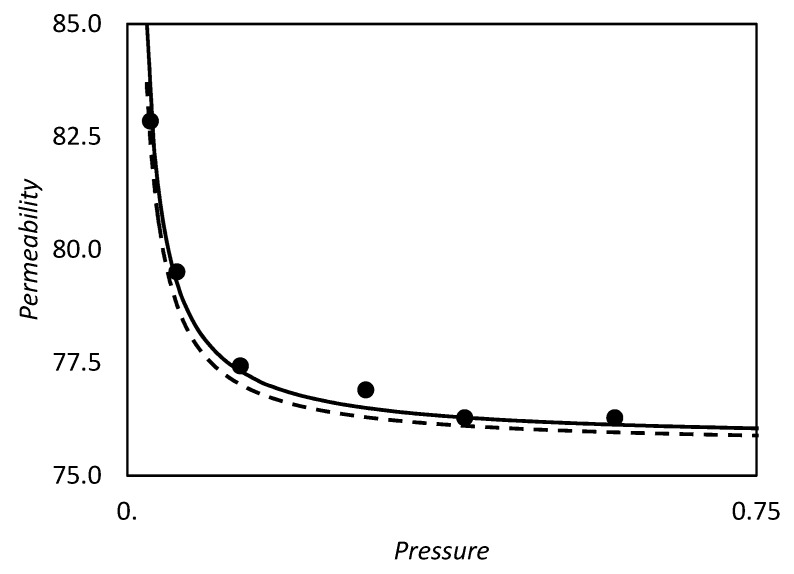
Oxygen permeability in a poly-dimethyl siloxane (PDMS) membrane with metallo-porphyrin fixed carrier. Circles are experimental data from Ohynagi et al. [26], *n* = 228 and *n* = 1 for continuous and broken line, respectively. Permeability is in (1 × 10^−9^ × cm^3^·cm/(cm^2^·s·atm)), pressure is in (atm).

Both the RC models [112,113] were tested successfully against the experimental data measured by Ohyanagi et al. [26] about the oxygen facilitated transport, Figure 16. As one can expect, the series RC circuits model provided the best agreement due to the presence of one additional parameter, the number of circuits, *n*, that, for the membranes case, represents the number of sublayers in which the thickness is divided, and can be calculated from the following:

$$n=N_{AV}C_{T}\left(\pi r_{s}L\right)$$

where $N_{AV}$ is the Avogadro number and $r_{s}$ is the kinetic radius of solute.

#### 3.1.7. Zarca et al., 2017. A Practical Approach to Fixed-Site Carrier Facilitated Transport Modeling for the Separation of Propylene/Propane Mixtures Through Silver-Containing Polymeric Membranes

Recently, in 2017, Zarca et al. [114] modeled the facilitated transport of propylene in silver salts, AgBF_4_ embedded in polymeric membranes (PVDF-HFP) with an analytical approach. Furthermore, that approach was extended to consider also the presence of mobile carriers inside the membranes [18] (see next paragraph).

The basic idea of the model proposed by Zarca et al. is that the free solute follows a pure physical diffusion mechanism while the complex diffusion is somehow related to the chemical reaction, similar to the dual mode based transport model of Noble [111]. Some differences arise when comparing the approach of Zarca et al. to the one of Noble. Noble considers four diffusion coefficients, while in this approach there are only two ones: the first one accounts for the pure physical diffusion mechanism, *D_dd_* in Noble’s notation, and the second one incorporates all the effects of the chemical reaction. The exchange between different zones of the membrane has been not considered explicitly, and a hopping mechanism was hypothesized.

The expression for the solute flux used by the authors is given hereafter, Equation (204):

(204) $$J_{A}=-D_{dd}\frac{dC_{A}}{dx}-A\frac{dC_{A}}{dx}$$

or in integrated form as:

(205) $$J_{A}=D_{dd}\frac{\left(C_{A}^{0}-C_{A}^{L}\right)}{L}+A\frac{\left(C_{A}^{0}-C_{A}^{L}\right)}{L}$$

where *A* is an effective diffusivity. Note that in both the above equations, the flux is given as function of the pure solute concentration gradient only. Considering the concentration on the boundaries in equilibrium with the gas phase, by definition of permeability, Equation (205) becomes:

(206) $$J_{A}=P\frac{\left(p_{A}^{0}-p_{A}^{L}\right)}{L}+K_{H}\frac{\left(p_{A}^{0}-p_{A}^{L}\right)}{L}$$

The effective permeability, or hopping constant as the authors called it, $K_{H}$ is the term which contains all the reaction effects. In the definition of this key parameter, Equation (207), an Arrhenius type of law was included to explicitly consider the temperature dependence of the facilitated transport mechanism:

(207) $$K_{H}=\alpha {\left(\frac{C_{T}}{1+K_{eq}C_{A}^{0}}\right)}^{\beta }e^{\frac{E_{A}}{R}\left(\frac{1}{293}-\frac{1}{T}\right)}$$

The first term in brackets is the free carrier concentration in chemical equilibrium with the upstream side concentration of free solute, $C_{A}^{0}$. In the original paper, the equilibrium constant was given in heterogeneous form accounting both for concentration (carrier and complex) and pressure (solute in gas phase). Here we have converted that expression in homogeneous terms and used concentrations only. However, the conversion between the two is straightforward.

**Figure 17:**
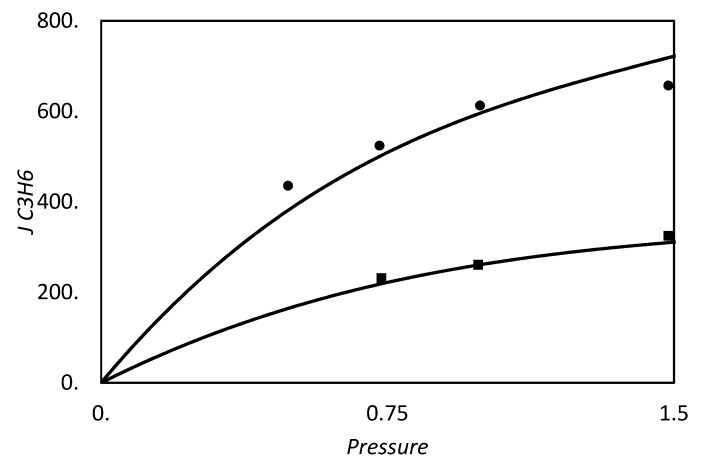
Ethylene flux as function of upstream pressure in membranes of poly-vinyldene fluoride (PVDF) containing AgBF_4_ as fixed carrier at two different temperatures. Symbols are experimental data from Zarca et al., lines are model results. From the top, *T* = 303.15 and 293.15 K. Flux is expressed in (1 × 10^−6^ mol/cm^2^·s), pressure is in (atm).

The model contains four parameters: $K_{eq}$ and $E_{A}$ (activation energy) which have a clear physical meaning, and α and β that are pure fitting parameters. In particular, β is a mathematical correction that allows the model to describe the percolation threshold limit. It was shown that the proposed method is in good agreement with the experimental data for facilitated transport of propylene in polymeric membranes containing silver ions, reported by the same authors, Figure 17. Its mathematical simplicity, coupled with the capability of fitting experimental data, in a larger range of experimental conditions than the one of Noble [111] based on the excess carrier hypothesis, makes this model a promising one for the facilitated transport systems.

#### 3.1.8. Zarca et al., 2017. Generalized Predictive Modeling for Facilitated Transport Membranes Accounting For Fixed and Mobile Carriers

As extension of the previous model, the authors included a third term in the flux expression to consider the case of hybrid fixed-mobile carriers systems [18].

Object of their study was the facilitated separation between propylene and propane but, in this case, an ionic liquid, BMImBF_4_, was also incorporated in the poly-vinyldene fluoride-co-hexafluoropropylene (PVDF-HFP) polymeric membranes, together with a silver salt, AgBF_4_. The resulting transport mechanism is a combination of the two effects of mobile and fixed carrier. Silver ions bounded to the polymer backbone act as fixed carriers while, the unbounded ones, due to the presence of liquid ion, are free to diffuse in the system, acting as mobile carrier. For these hybrid systems the total solute flux has the form:

(208) $$J_{A}=P\frac{\left(p_{A}^{0}-p_{A}^{L}\right)}{L}+K_{H}\frac{\left(p_{A}^{0}-p_{A}^{L}\right)}{L}+P_{comp}\frac{\left(p_{A}^{0}-p_{A}^{L}\right)}{L}$$

where $K_{H}$ is the enhanced permeability related to fixed sites, given by Equation (207) and $P_{comp}$ is the additive permeation term accounting for the presence of mobile carrier facilitation. Since in mobile facilitated transport the percolation limit does not exist, the parameter accounting for that in Equation (207), β, is set equal to one.

Following a kind of solution–diffusion model, $P_{comp}$ is calculated as the product of the chemical induced solubility, by means of chemical reaction in liquid phase with free silver ions, and the diffusion coefficient of the solute-complex in the ionic liquid.

The solubility induced by the chemical reaction has been derived from the chemical equilibrium as the concentration of the complexed species divided by the upstream partial pressure of solute A. To do that, the authors considered that the free solute concentration in the liquid phase could be explained by the Henry’s law using the upstream partial pressure, so that the equilibrium complex concentration is given by:

(209) $$C_{AC}=\frac{K_{eq}^{\prime }C_{T}Hp_{A}^{0}}{1+K_{eq}^{\prime }Hp_{A}^{0}}$$

where H is the Henry’s constant and $K_{eq}^{\prime }$ is the chemical equilibrium constant in the liquid environment. The solubility coefficient, $S_{AC}$, is obtained by dividing Equation (209) for the upstream partial pressure and is shown in Equation (210).

(210) $$S_{AC}=\frac{K_{eq}^{\prime }C_{T}H}{1+K_{eq}^{\prime }Hp_{A}^{0}}$$

In the end, the permeability through the mobile carrier mechanism is:

(211) $$P_{comp}=\frac{K_{eq}^{\prime }C_{T}H}{1+K_{eq}^{\prime }Hp_{A}^{0}}D_{comp}$$

where $D_{comp}$ indicates the diffusion coefficient of the complex species in the ionic liquid environment. Arrhenius–like functions were used to describe the thermal dependence of the equilibrium constant, $K_{eq}^{\prime }$, of the diffusion coefficient and of the Henry’s constant, so that the present model could in principle describe facilitation at different temperatures.

**Figure 18:**
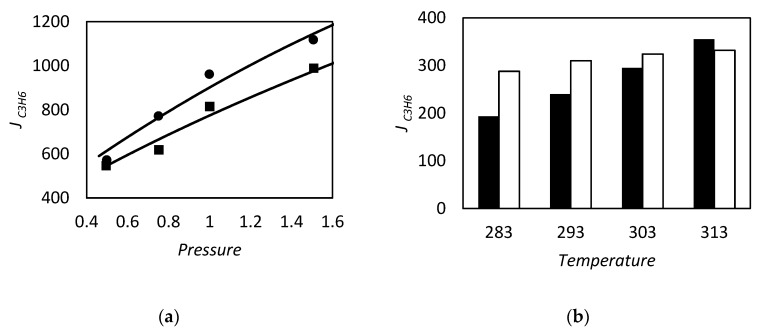
Ethylene flux in polymeric membranes (PVDF-HFP)/BMImBF_4_/AgBF_4_. (**a**) Influence of ethylene pressure at two different temperature. Symbols are experimental data, lines are model calculations from Zarca et al. [18]. From the top, *T* = 303.15 K, 293.15 K; (**b**) Prediction of fixed site (black) and mobile carrier (white) contribution to total flux as function of temperature. Flux is expressed in (1 × 10^−6^ mol/cm^2^·s), pressure is in (atm), temperature is in (K).

The only pure fitting parameter is α, since β is set equal to one a priori in this case. The experimental data for the system under study were modeled with satisfactory results, as we can see in Figure 18a.

Furthermore, the temperature dependence relations and the co-presence of different terms for the two distinct facilitation mechanisms, allow the model to predict the experimental conditions for which one effect overcomes the other, as shown in Figure 18b, giving interesting and practical information.

Even if containing fitting parameters, the method proposed by Zarca et al. seems to be a useful calculation tool for hybrid facilitated carrier membranes performance.

## 4. Conclusions

In this work, different mathematical approaches developed to tackle the mass transfer problem in facilitated transport membranes are reviewed, and their range of applicability and accuracy analyzed for both mobile and fixed carrier systems.

In particular, the analysis is carried out for a simplified case where only one reaction occurs between the carrier and the target permeants. Even for this simplified system, there is no general analytical solution, so that most modeling approaches are based on simplified solutions approximating the real membrane behavior.

Two opposite transport regimes define the limiting cases of such systems: the pure physical diffusion of unreacted solute and the chemical equilibrium regime. Both scenarios are oversimplifications of the problem and lead to unreliable quantitative results in most operative conditions. Nevertheless, such conditions can be used as reference states to develop approximate methods for the solution of the facilitated transport problem.

In this direction, for the mobile carrier case, Goddard et al. [84] divided the membrane thickness in three sublayers, assuming the chemical equilibrium in the middle while, in the adjacent boundary zones, the same order of magnitude was considered for the diffusion and reaction mechanisms.

Smith et al. [88] studied the transport in ‘thin’ or ‘thick’ films considering that, near the boundaries, the reaction should not impact the solute transport, compared to the inner core while Kreuzer and Hoofd derived their solution [86,87], considering that the species concentrations deviate from the equilibrium ones by a position departure function which goes quickly to zero as the distance from boundaries increase.

The same approach was also used by Teramoto [74] and Morales-Cabrera et al. [98], and led to the conclusion that for Damköhler number ≫1, the internal equilibrium core is a reasonable assumption. Moreover, the last two methods are also able to predict the presence of an optimal range of equilibrium constants that maximize the facilitation effect when parameters such as species diffusivities, solute partial pressure or concentration change.

In conclusion, among the models reported in this work for the mobile carrier case, the ones from Teramoto [74] and from Morales-Cabrera et al. [98], seem the more flexible ones as they can be applied in a wide range of operative conditions, spacing from the pure physical to chemical equilibrium regime, without the assumption of equal diffusivity of carrier and reaction product. However, they consist in a system of equations that need to be solved by using a numerical technique and it can be not straightforward.

In cases for which the equality between carrier and complex diffusivities is reasonable, and if the excess of carrier concentration can be assumed, the simple models provided by Smith and Quinn [93], Noble et al. [72] and Jeema and Noble [96], seem to be the best choice due to their descriptive ability coupled with a very simple mathematical form.

In the fixed carrier systems, the only species that can freely diffuse across the film are, obviously, the solute of interest and the other dissolved species, since both the carrier and the complex (reaction product) are immobilized in the matrix and can only vibrate around their equilibrium position. It follows that the chemical reaction does not solely enhance the overall solute solubility in the systems, but acts as a catalyst in the diffusion process.

Noble [111] formalized this effect by considering all the possible diffusion paths accessible by the solute in the solid matrix, starting from the generalized dual mode theory of Barrer [108]. He concluded that, in principle, a facilitation may be detected even if the carrier sites are too far one from another and a pure hopping mechanism is not allowed, contrarily for example to what foreseen by Cussler et al. in their modeling approach [100]. Another important result from Noble’s work was that fixed carrier facilitated transport systems can be described by a simple modeling approach, mathematically equivalent to that developed by Smith and Quinn for mobile carrier systems [93].

Other interesting approaches in the analysis of fixed carrier systems comes from the analogy with RC parallel circuit model provided by Kang et al. [112,113] and from the description recently proposed by Zarca et al. for pure fixed sites, [114] and for the hybrid cases (mobile + fixed carriers), [18]. The resulting models are based on the use of some fitting parameters but, nevertheless, seem to be a very useful tool to indagate these peculiar systems.

This work shows that there are many possible tools that can be used to describe and understand facilitated transport membrane and can be seen as a valid support in the development of facilitated transport membranes and in the correct exploitation of the already existing ones.

However, it is worth to mentioning that for both the mobile and fixed carrier systems one of the main issue is the knowledge of the physical and chemical parameters needed to quantitatively describe the transport. In many cases, the experimental determination of these latter is not so easy, in particular for the fixed carrier cases. To overcome this point, the use of advanced simulations by the molecular dynamics technique and/or the use of advanced thermodynamic models, or equation of state, that can describe complex multicomponent systems starting from the sole knowledge of the pure components behavior, could be a way to open new windows in this modeling field.

## Figures and Tables

**Table 1:** Analogies between electrons transport and mass transport in resistor–capacitor circuits (RC) parallel circuit and facilitated transport systems with fixed sites carrier [112].

	RC circuit	Facilitated Systems
**Flux**	$j_{e}=\sigma \frac{\Delta V}{L}$	$J=P\frac{p^{0}}{L}$
**Driving Force**	$\frac{\Delta V}{L}$	$\frac{p^{0}}{L}$
**Proportionality**	σ	P
**Capacitor Effect**	q=CV	$C_{AC}=\frac{C_{T}C_{A}K_{eq}}{1+C_{A}K_{eq}}$

## Supplementary Materials

The following are available online at http://www.mdpi.com/2077-0375/9/2/26/s1, S1: Supplementary information reporting additional details on the derivation of the following models: Goddard, Shultz and Bassett, 1969, On membrane diffusion with near equilibrium reaction. Kreuzer and Hoofd, 1970, Facilitated diffusion of oxygen in the presence of hemoglobin. Yung and Probstein, 1973, Similarity considerations in facilitated transport. Smith, Meldon, Colton, 1973, An analysis of carrier facilitated transport. Basaran et al., 1989, Facilitated Transport with Unequal Carrier and Complex Diffusivities. Morales-Cabrera et al., 2002, Approximate Method for The Solution of Facilitated Transport Problems in Liquid Membranes.
