# Supplementary material for: Models for Facilitated Transport Membranes: A Review
Source: Membranes (Basel). 2019 Feb 2;9(2):26. doi: 10.3390/membranes9020026 (PMC6409752; doi:10.3390/membranes9020026)
Supplement: Supplementary file 1 [file membranes-09-00026-s001.pdf]

Supplementary Materials: Models for Facilitated Transport Membranes: A Review

R. Rea, M.G. De Angelis, M. Giacinti Baschetti

Goddard, Shultz and Bassett, 1969, On membrane diffusion with near equilibrium reaction.

$$G(Z) = \sqrt{\frac{Z^5}{1+Z^2}} \tag{S1}$$

$$\overline{Z} = \frac{\sigma}{1+K_{eq}C_A^0} \tag{S2}$$

$$\underline{Z} = \frac{\sigma}{1+K_{eq}C_A^L} \tag{S3}$$

$$\sigma = K_{eq}C_T\frac{D_{AC}}{D_A} \tag{S4}$$

Kreuzer and Hoofd, 1970, Facilitated diffusion of oxygen in the presence of hemoglobin.

$$E_1 = \frac{1}{2\alpha^2 D_{AC}D_A}\left(\frac{k_f C_C^0}{D_A}\frac{J_A}{\alpha} + k_r C_{AC}^0 - k_f C_A^0 C_C^0\right) \tag{S5}$$

$$H_1 = \frac{1}{2\beta^2 D_{AC}D_A}\left(\frac{k_f C_C^L}{D_A}\frac{J_A}{\beta} + k_r C_{AC}^L - k_f C_A^L C_C^L\right) \tag{S6}$$

Yung and Probstein, 1973, Similarity considerations in facilitated transport.

$$\alpha = \frac{C_T}{C_C^0} \tag{S7}$$

$$\eta = \frac{\left(\frac{C_C}{C_C^0} - 1\right)}{\kappa} \tag{S8}$$

$$\eta_L = \frac{\left(\frac{C_C^L}{C_C^0} - 1\right)}{\kappa} \tag{S9}$$

$$\Delta = \frac{D_{AC}}{D_A}K_{eq}C_T \tag{S10}$$

$$\begin{aligned}\eta_1 = B&\left[1 + \sum_{n=1}^{\infty}\frac{\varepsilon^{3n}}{(3n)(3n-1)(3n-3)(3n-4)\times\ldots 3\times 2}\right]\\ +C&\left[\varepsilon + \sum_{n=1}^{\infty}\frac{\varepsilon^{3n+1}}{(3n)(3n+1)(3n-2)(3n-3)\times\ldots 3\times 4}\right]\end{aligned} \tag{S11}$$

B, C are two expansion coefficients to be determined by BC's.

$$\beta = \kappa^{-1}\varepsilon - \alpha\epsilon^{-1/3}\kappa^{-2/5} \tag{S12}$$

$$\varepsilon = [1+\kappa(1-x')](\epsilon\kappa^2)^{-1/3} \tag{S13}$$

Smith, Meldon, Colton, 1973, An analysis of carrier facilitated transport.

$$J_{A,linearized}^{*} = \frac{1}{1+\left(F_{eq}-1\right)\left[\Upsilon\left(\frac{\widetilde{\lambda_0}}{L}\right) + \frac{1}{\Upsilon}\left(\frac{\widetilde{\lambda_L}}{L}\right)\right]} \tag{S14}$$

$$\Upsilon = \frac{k_f C_A^L + k_r}{k_f C_A^0 + k_r} \tag{S15}$$

$$G_0(0) - 2N_1\left(\frac{\lambda_0}{\widetilde{\lambda_0}}\right)^8 (J_A^*)^2 + \frac{1}{3}N_1N_2\left(\frac{\lambda_0}{\widetilde{\lambda_0}}\right)^2 G_0^2(0)$$
$$+\frac{1}{4}N_1\left\{1 - \frac{N_2}{[1 - N_3G_0(0)]^2}\right\}\left\{[1 - N_3G_0(0)]\left(\frac{\lambda_0}{\widetilde{\lambda_0}}\right)^5 J_A^*G_0(0)\right\} = \left(\frac{\lambda_0}{\widetilde{\lambda_0}}\right)^3 J_A^*\frac{1}{[1 - N_3G_0(0)]} \quad \text{(S16)}$$

$$G_L(0) + 2M_1\left(\frac{\lambda_L}{\widetilde{\lambda_L}}\right)^8 (J_A^*)^2 - \frac{1}{3}M_1M_2\left(\frac{\lambda_L}{\widetilde{\lambda_L}}\right)^2 G_L^2(0)$$
$$-\frac{1}{4}M_1\left\{1 - \frac{M_2}{[1 + M_3G_L(0)]^2}\right\}\left\{[1 + M_3G_0(0)]\left(\frac{\lambda_L}{\widetilde{\lambda_L}}\right)^5 J_A^*G_L(0)\right\} = \left(\frac{\lambda_L}{\widetilde{\lambda_L}}\right)^3 J_A^*\frac{1}{[1 + M_3G_L(0)]} \quad \text{(S17)}$$

$$N_1 = k_f\widetilde{\lambda_0}^5[C_A^0 - C_A^L][1 + F_{eq}][k_fC_A^0 + k_r]/(D_{AC}{}^2L) \quad \text{(S18)}$$

$$N_2 = {D_Ak_fk_rC_T}\Big/{D_A\left(k_fC_A^0 + k_r\right)^2} \quad \text{(S19)}$$

$$N_3 = {k_f^2k_rC_T\widetilde{\lambda_0}^3[C_A^0 - C_A^L][1 + F_{eq}]}\Big/{D_A\left(k_fC_A^0 + k_r\right)^2L} \quad \text{(S20)}$$

$$N_4 = {k_f^2k_rC_T\widetilde{\lambda_0}^3[1 + F_{eq}]}\Big/{D_A\left(k_fC_A^0 + k_r\right)L} \quad \text{(S21)}$$

$$M_1 = k_f\widetilde{\lambda_L}^5[C_A^0 - C_A^L][1 + F_{eq}][k_fC_A^L + k_r]/(D_{AC}{}^2L) \quad \text{(S22)}$$

$$M_2 = {D_{AC}k_fk_rC_T}\Big/{D_A\left(k_fC_A^L + k_r\right)^2} \quad \text{(S23)}$$

$$M_3 = {k_f^2k_rC_T\widetilde{\lambda_L}^3[C_A^0 - C_A^L][1 + F_{eq}]}\Big/{D_A\left(k_fC_A^L + k_r\right)^2L} \quad \text{(S24)}$$

$$M_4 = {k_f^2k_rC_T\widetilde{\lambda_L}^3[1 + F_{eq}]}\Big/{D_A\left(k_fC_A^L + k_r\right)L} \quad \text{(S25)}$$

Basaran et al., 1989, Facilitated Transport with Unequal Carrier and Complex Diffusivities.

$$\delta = \frac{D_{AC}}{D_A} \quad \text{(S26)}$$

$$\sigma = \frac{1}{KT} \quad \text{(S27)}$$

$$\gamma_0 = \frac{1}{T} \quad \text{(S28)}$$

$$\gamma_1 = \frac{\overline{C_A^L}}{T} \quad \text{(S29)}$$

$$\eta_0 = \sigma + d\gamma_0 \quad \text{(S30)}$$

$$\eta_1 = \sigma + d\gamma_1 \quad \text{(S31)}$$

$$\omega = (ln(\eta_0) - ln(\eta_1))(\eta_0 - \eta_1)^{-1} \quad \text{(S32)}$$

$$p_0 = -\sigma\delta\frac{\sigma^2 + \gamma_0\gamma_1 + (\gamma_0+\gamma_1)\sigma}{\eta_0\eta_1} \quad \text{(S33)}$$

$$p_1 = \sigma\delta - \eta_0\eta_1 \quad \text{(S34)}$$

$$p_2 = \eta_0\eta_1 \quad \text{(S35)}$$

$$\rho = \frac{(\eta_0{}^{-1} + \eta_1{}^{-1})}{2} \quad \text{(S36)}$$

Morales-Cabrera et al., 2002, Approximate Method for The Solution of Facilitated Transport Problems in Liquid Membranes.

$$A_L = \left[\frac{\varphi_R}{\varphi_L tanh(\varphi_R) + \varphi_R tanh(\varphi_L)}\right]\left[\left(\overline{C_A^1} + \frac{\alpha_R}{{\varphi_R}^2} + \frac{\beta_R}{{\varphi_R}^2}\right)\frac{1}{cosh(\varphi_R)} - \left(1 - \frac{\alpha_L}{{\varphi_L}^2} - \frac{\alpha_R}{{\varphi_R}^2}\right)\frac{tanh(\varphi_R)}{\varphi_R} + \frac{\beta_L}{{\varphi_L}^2} - \frac{\beta_R}{{\varphi_R}^2}\right] \quad \text{(S37)}$$

$$A_R = \frac{\varphi_L}{\varphi_R} A_L - \frac{1}{\varphi_R}\frac{\alpha_L}{{\varphi_L}^2} + \frac{\alpha_R}{{\varphi_R}^2} \quad \text{(S38)}$$

$$B_L = \frac{1}{cosh(\varphi_L)}\left[A_L sinh(\varphi_L) + \left(1 - \frac{\alpha_L}{{\varphi_L}^2} + \frac{\beta_L}{{\varphi_L}^2}\right)\right] \quad \text{(S39)}$$

$$B_R = \frac{1}{cosh(\varphi_R)}\left[-A_R sinh(\varphi_R) + \left(\overline{C_A^1} + \frac{\alpha_R}{{\varphi_R}^2} + \frac{\beta_R}{{\varphi_R}^2}\right)\right] \quad \text{(S40)}$$

$$\alpha_L = \phi^2\left[\left(\frac{1}{r_C} + \frac{1}{Kr_{AC}}\right)\overline{J_A}\right] \quad \text{(S41)}$$

$$\alpha_R = \phi^2\left[\left(\frac{\overline{C_A^1}}{r_C} + \frac{1}{Kr_{AC}}\right)\overline{J_A}\right] \quad \text{(S42)}$$

$$\beta_L = -\phi^2\left[\overline{C_C^{-1}} + \frac{T_C}{r_C} + \frac{T_{AC}}{Kr_{AC}}\right] \quad \text{(S43)}$$

$$\beta_R = -\phi^2\left[\overline{C_A^1 C_C^1} + \frac{\overline{C_A^1}T_C}{r_C} + \frac{T_{AC}}{Kr_{AC}}\right] \quad \text{(S44)}$$

$$r_{AC} = \frac{D_{AC}}{D_A} \quad \text{(S45)}$$

$$r_C = \frac{D_C}{D_A} \quad \text{(S46)}$$
